# Cryo-electron microscopy structure and translocation mechanism of the crenarchaeal ribosome

**DOI:** 10.1093/nar/gkad661

**Published:** 2023-08-22

**Authors:** Ying-Hui Wang, Hong Dai, Ling Zhang, Yun Wu, Jingfen Wang, Chen Wang, Cai-Huang Xu, Hai Hou, Bing Yang, Yongqun Zhu, Xing Zhang, Jie Zhou

**Affiliations:** Life Sciences Institute, Zhejiang University, Hangzhou, Zhejiang 310058, China; Life Sciences Institute, Zhejiang University, Hangzhou, Zhejiang 310058, China; Life Sciences Institute, Zhejiang University, Hangzhou, Zhejiang 310058, China; Life Sciences Institute, Zhejiang University, Hangzhou, Zhejiang 310058, China; Center for Cryo-Electron Microscopy, Zhejiang University School of Medicine, Hangzhou, Zhejiang 310058, China; Department of Pathology of Sir Run Run Shaw Hospital, and Department of Biophysics, Zhejiang University School of Medicine, Hangzhou, Zhejiang 310058, China; Center for Cryo-Electron Microscopy, Zhejiang University School of Medicine, Hangzhou, Zhejiang 310058, China; Department of Pathology of Sir Run Run Shaw Hospital, and Department of Biophysics, Zhejiang University School of Medicine, Hangzhou, Zhejiang 310058, China; Center for Cryo-Electron Microscopy, Zhejiang University School of Medicine, Hangzhou, Zhejiang 310058, China; Department of Pathology of Sir Run Run Shaw Hospital, and Department of Biophysics, Zhejiang University School of Medicine, Hangzhou, Zhejiang 310058, China; Institute of Medical Research, Northwestern Polytechnical University, Xi’an, Shaanxi 710072, China; Life Sciences Institute, Zhejiang University, Hangzhou, Zhejiang 310058, China; Life Sciences Institute, Zhejiang University, Hangzhou, Zhejiang 310058, China; Center for Cryo-Electron Microscopy, Zhejiang University School of Medicine, Hangzhou, Zhejiang 310058, China; Department of Pathology of Sir Run Run Shaw Hospital, and Department of Biophysics, Zhejiang University School of Medicine, Hangzhou, Zhejiang 310058, China; Life Sciences Institute, Zhejiang University, Hangzhou, Zhejiang 310058, China

## Abstract

Archaeal ribosomes have many domain-specific features; however, our understanding of these structures is limited. We present 10 cryo-electron microscopy (cryo-EM) structures of the archaeal ribosome from crenarchaeota *Sulfolobus acidocaldarius* (*Sac*) at 2.7–5.7 Å resolution. We observed unstable conformations of H68 and h44 of ribosomal RNA (rRNA) in the subunit structures, which may interfere with subunit association. These subunit structures provided models for 12 rRNA expansion segments and 3 novel r-proteins. Furthermore, the 50S–aRF1 complex structure showed the unique domain orientation of aRF1, possibly explaining P-site transfer RNA (tRNA) release after translation termination. *Sac* 70S complexes were captured in seven distinct steps of the tRNA translocation reaction, confirming conserved structural features during archaeal ribosome translocation. In aEF2-engaged 70S ribosome complexes, 3D classification of cryo-EM data based on 30S head domain identified two new translocation intermediates with 30S head domain tilted 5–6° enabling its disengagement from the translocated tRNA and its release post-translocation. Additionally, we observed conformational changes to aEF2 during ribosome binding and switching from three different states. Our structural and biochemical data provide new insights into archaeal translation and ribosome translocation.

## Graphical Abstract

**Graphical Abstract:**
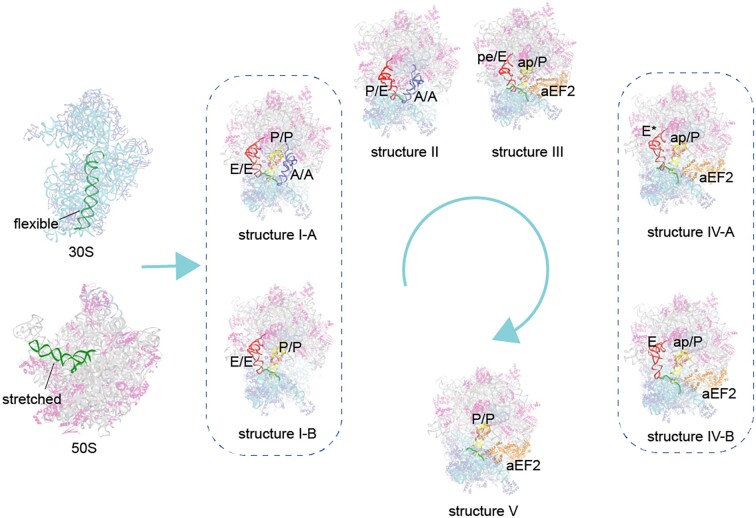

## INTRODUCTION

The ribosome is a large macromolecular machine that synthesizes proteins in all living cells. Although the structures of many bacterial and eukaryotic ribosomes are known (1–5), a few are described in archaea (and are for euryarchaeota) (3,6–11). The eocyte hypothesis proposes that eukaryotes emerged from crenarchaeota (12); however, high-resolution structures of the crenarchaeal ribosome are lacking. The archaeal ribosome is similar to that of eukaryotes in many aspects. For example, the archaeal ribosome contains ribosomal r-proteins that are universally specific to eukarya and archaea but not bacteria (13,14). Understanding the crenarchaeal ribosome structure can provide insights into eukaryotic ribosome evolution. Furthermore, because archaea thrive in extreme conditions (15), the ribosomal RNA (rRNA) of the archaeal ribosome at key regions (such as the peptidyl transferase center) was shown to confer stabilization of the ribosome at high temperatures (16). High-resolution structures of crenarchaeal ribosome functional complexes may enrich our understanding of ribosomal rRNA folding [such as expansion segments (ESs)]. Moreover, it may clarify differences with other species regarding subunit association and transfer RNA (tRNA) binding.

tRNA and messenger RNA (mRNA) translocation through the ribosome, catalyzed by the elongation factor aEF2 in archaea (known as EF-G and eEF2 in bacteria and eukarya, respectively), is an important and complex step during translation (17–22). Despite extensive research on bacterial and eukaryotic ribosome translocation, research on archaeal ribosome translocation is lagging. Studies on bacteria have revealed that tRNAs first move along the 50S subunit during translocation in P/E and A/P hybrid states (23–35). Subsequently, the tRNA anticodon stem loop (ASL)-associated mRNA moves on the 30S subunit (36–41). The last step involves a large-scale rotation of the 30S head domain and formation of ap/P and pe/E chimeric hybrid state tRNAs, still in contact with the 30S head domain (29,33–35,39,42–50). However, the next step, where the 30S head domain releases tRNAs and prevents tRNA slippage during reverse swiveling of the 30S head in the late stages of translocation, is not fully understood. In bacteria, domain IV of EF-G plays the doorstop role and maintains tRNAs and mRNA in the post-translocation (POST) position (33,35,37,38,45,48). Notably, the ribosome may work with translocase to achieve the POST state. Since the mechanisms of ribosome translocation are conserved across evolution, research on archaeal translocation may answer unresolved questions about this issue.

To gain insights into the archaeal ribosome architecture and translocation, we obtained cryo-electron microscopy (cryo-EM) structures of three *Sulfolobus acidocaldarius* (*Sac*) ribosome subunits and seven *Sac* 70S·tRNA_2_·mRNA or *Sac* 70S·tRNA_2_·mRNA·aEF2·GDPNP complexes at resolutions of 2.7–5.7 Å. These structures illustrate details of crenarchaeal ribosome architecture. By comparing with structures of bacterial translocation complexes, we identified new translocation intermediates with tilted 30S head domain conformation.

## MATERIALS AND METHODS

### Ribosome purification from *Sac* DSM639

*Sac* (DSM639) ribosomes were prepared following a previously described protocol (51); this protocol can yield tightly coupled 70S ribosomes in bacteria, such as *Escherichia coli*, or euryarchaeota, such as *Pyrococcus furiosus* (7). However, the sedimentation diagram of the sucrose gradient showed that only the 50S and 30S ribosome subunits could be obtained in the *Sac* strain. First, *Sac* DSM639 cells were grown at 75°C and pH 3.5. Next, to obtain *Sac* ribosomes, 3 g cell pellets were harvested and dissolved in 30 ml of buffer A (25 mM Tris–HCl, pH 7.5, 100 mM NH_4_Cl and 10.5 mM MgCl_2_) at 4°C, lysed (high-pressure cell disruption equipment name: ConstantSystem, 18 kPa) and centrifuged at 18 000 rpm for 40 min. Afterward, the supernatant was loaded onto a 37.7% sucrose cushion (20 mM Tris–HCl, pH 7.5, 37.7% sucrose, 100 mM NH_4_Cl and 10.5 mM MgCl_2_) and centrifuged in a Ti45 rotor (Beckman) at 38 000 rpm for 21 h at 4°C. Subsequently, the pellet was suspended in buffer A, loaded onto a 10–35% sucrose gradient and spun in an SW32 rotor (Beckman) at 20 000 rpm for 13 h at 4°C. Fractions containing 30S and 50S subunits were collected using Biocomp 152 Piston Gradient Fractions and pelleted separately after centrifugation in a Ti70 rotor (Beckman) at 38 000 rpm for 17 h at 4°C. Lastly, pellets were suspended in buffer B (25 mM HEPES K, pH 7.5, 30 mM KCl, 10 mM MgCl_2_ and 1 mM dithiothreitol), flash-frozen in liquid nitrogen and stored at −80°C until further use.

### Expression and purification of aEF2, aRF1 and aEF1A

*Sac* DSM639 genes encoding aEF2, aRF1 and aEF1A were cloned into the pET21b vector (Novagen) under the control of a T7 promoter with C-terminal 6xHis tag and expressed in the *E. coli* BL21 strain. Next, the cells were harvested after induction using 1 mM isopropyl β-d-1-thiogalactopyranoside for 3 h at 37°C. Afterward, the cells were suspended in 50 ml lysis buffer (50 mM Tris–HCl, pH 8.0, 150 mM KCl, 5 mM iminazole and 5% glycerol). Next, resuspended cell pellets were lysed, and the debris was removed via centrifugation. Subsequently, the lysate was incubated at 70°C for 10 min and centrifuged to remove endogenous *E. coli* proteins. Afterward, the supernatant was passed through a Ni-NTA column (QIAGEN). Elutions of aEF2, aRF1 and aEF1A were purified using a 5 ml HiTrap Q HP column (GE Healthcare) equilibrated in buffer containing 25 mM Tris–HCl (pH 8.0), 500 mM KCl and 1 mM dithiothreitol. The aEF2 fraction was diluted with buffer A (25 mM Tris–HCl, pH 8.0, 200 mM KCl and 1 mM dithiothreitol) and concentrated by ultrafiltration to >20 mg/ml. The aRF1 and aEF1A fractions were diluted in buffer A and concentrated to 40 and 13 mg/ml, respectively. Lastly, the purified samples were flash-frozen in liquid nitrogen and stored at −80°C.

### tRNA, mRNA and S-100 preparation

tRNA^phe^ was overexpressed and purified as described in (52). For mRNA preparation, the DNA sequence containing the Shine–Dalgarno (SD) sequence (53) and a linker containing the Phe codon [5′-GAAAUUAAUACGACUCACUAUAGGUGAGGUGAUCC(UUU)_6_-3′ for elongation complexes or 5′-GAAAUUAAUACGACUCACUAUAGGUGAGGUGAUCC(UUU)_6_UAA-3′ for the termination complex] was synthesized using Integrated DNA Technologies. Next, the synthesized DNA templates were transcribed *in vitro* using T7 RNA polymerase and purified using a preparative urea–polyacrylamide gel electrophoresis (9.5% polyacrylamide and 1× TBE, 7 M urea). Subsequently, the mRNA was extracted using phenol–chloroform and precipitated with 100% ethanol. Lastly, the purified tRNA and mRNA samples were flash-frozen and stored at −80°C for further use. A cellular enzyme extract (S-100) was used for tRNA^Phe^ aminoacylation and prepared from MRE600 *E. coli* as described (54).

### Formation of an aRF1–50S complex

For ribosome·aRF1 complex formation, 40 pmol 30S ribosome, 40 pmol 50S ribosome, 1600 pmol mRNA and 600 pmol tRNA^Phe^ were incubated at 70°C for 20 min in buffer containing 20 mM HEPES K (pH 7.5), 18 mM MgCl_2_, 10 mM NH_4_Cl and 3 mM spermine (Sigma). Subsequently, 400 pmol aRF1 (10-fold), 400 pmol aEF1A (10-fold) and 0.5 mM GTP were added to the reaction solution and incubated for 20 min at 70°C. Next, the mixtures were loaded onto a 37.7% sucrose cushion in reaction buffer. After ultracentrifugation (100 000 rpm for 30 min), the pellet was analyzed using sodium dodecylsulfate–polyacrylamide gel electrophoresis (SDS–PAGE) gel to determine aRF1’s binding efficiency. The results indicated that only aRF1 could bind to the 50S ribosome.

### *Sac* 70S ribosomal complex formation

As indicated by analytical sucrose gradient centrifugation, a few 70S ribosomes could be detected at high magnesium ion (Mg^2+^) concentrations. We initially tried to obtain 70S *Sac* ribosomes by collecting the fractions from the later part of the 50S peak. However, we could not find 70S particles after pelleting these ribosomes and applying cryo-EM analysis. Reportedly, 70S ribosomal complexes can only be obtained in active translation (55). According to a previously reported *in vitro* biochemical assay, the *Sac*70S ribosomal complex can be obtained in a polyuridine-directed cell-free system (51). Therefore, we performed complex formation experiments by adding the ligands in the appropriate buffer. For ribosome·tRNA^Phe^·mRNA complex formation, we mixed ribosomal 50S and 30S subunits from *Sac* and incubated with Phe-tRNA^Phe^ and mRNA in a buffer containing spermine and high Mg^2+^ (15–20 mM) but with low cation concentration (<30 mM K^+^ and <10 mM NH_4_^+^) (56). Moreover, we incubated 30 pmol 30S ribosome, 60 pmol 50S ribosome, 1600 pmol mRNA (specially designed with a strong SD sequence) and 700 pmol tRNA^Phe^ at 70°C for 20 min in 10 μl reaction system. To prepare aEF2·GDPNP·70S·tRNA·mRNA complex, 600 pmol (10-fold) aEF2 and GDPNP (final concentration, 1 mM) pre-incubated at room temperature for 10 min were added to the 70S ribosome·tRNA^Phe^·mRNA complexes for further incubation for 20 min at 70°C. Next, the reaction mixtures were loaded onto a 37.7% sucrose cushion in optimal buffer conditions. After ultracentrifugation (100 000 rpm for 30 min), the pellet was analyzed using SDS–PAGE gels to determine the binding efficiency of translation factors, such as aEF2. The gel results indicated that aEF2 could bind to *Sac* ribosomes (Supplementary Figure S1B).

### Cryo-EM data acquisition

Samples 1–4 were incubated with 2% trehalose to protect the samples from damage during the freezing and improve the resolution of the cryo-EM images. Quantifoil holey carbon grids (R1.2/1.3, 300 mesh, Au, Germany) were glow-discharged at 20 mA for 180 s. Next, Vitrobot Mark IV (FEI, Hillsboro, Oregon) was pre-equilibrated to ∼22°C with 100% humidity for 30 min before plunging. Afterward, 4 μl of ∼5 mg/ml 70S ribosomal complex was applied to each grid and flash-frozen in liquid nitrogen-cooled liquid ethane with blotting for 6 s. Subsequently, the grids were imaged with a Titan Krios (FEI, D3798, Hillsboro, Oregon) electron microscope operated at 300 keV and equipped with a K3 Summit detector (Gatan, Warrendale, PA) and GIF Quantum energy filter. Movie stacks were automatically collected using AutoEMation (written by Jianlin Lei), and images were recorded at 81 000× magnification with a defocus range of −1.5 to −2.0 μm. Afterward, each stack was exposed for 2.56 s, with an exposure time of 0.08 s per frame, resulting in 32 frames per stack. Lastly, the total dose rate was ∼50 e^−^/Å^2^ for each stack, and the stacks were motion-corrected using MotionCor2 (57) and binned 2-fold, resulting in a pixel size of 1.087 Å/pixel.

### Image processing

Detailed data processing is described in the Supplementary Data. Movies were aligned with MotionCor2 (57), and the CTF was estimated with CTFFIND 4.1.8 (58). Moreover, particles were picked with crYOLO using the general model (59), which was trained on low-pass filtered images. Additionally, particles were initially extracted with a box size of 380 pixels and a pixel size of 1.087 Å in RELION. The subsequent processing was performed in RELION 3.1.2 (60) and cryoSPARC v4.0 (61). The 2D classification was performed using cryoSPARC (61). Next, the 70S *Sac* ribosome particle classes were selected and subjected to *ab initio* reconstruction to obtain the initial model using cryoSPARC. Afterward, 3D classification was performed in RELION with (for 70S complexes) or without (for focused classification) angular sampling. For focused classification with partial signal subtraction, docking high-resolution *Sac* 30S subunit or the 30 subunit head domain was combined with the commands ‘color zone’, ‘split map’ and volume eraser in UCSF Chimera (62) to create volumes for generating masks. Lastly, classification by a focused mask was performed using cryoSPARC v4.0 to identify structures I-A and I-B or structures IV-A and IV-B. The procedure of calibrating the pixel size for the maps consists of cross-correlation calculations between the cryo-EM map and a map calculated from the atomic models. For 50S large subunit (LSU; PDB: 1FFK) and for varying scales by increments of 0.01 of the pixel size (in Chimera software, use map simulated from atoms, resolution 3.5 Å), and in the range of 1.047–1.087, we got correlation of 0.5316, 0.5985, 0.6136, 0.563 and 0.4794, respectively, on the data (nominal pixel size of 1.087 Å), with 1.067 Å being the optimal value. For 70S (PDB: 4V6F), and for the range of 1.047–1.087, we got correlation of 0.7232, 0.7342, 0.7371, 0.7347 and 0.7175, respectively, on the data (nominal pixel size of 1.087 Å), with 1.077 Å being the optimal value.

### Model building and refinement

The detailed model-building process is described in the Supplementary Data. The crystal structures of *Haloarcula marismortui* (*Hma*)’s 50S LSU (PDB ID: 1FFK) and *Thermus thermophilus* (*Tth*) 30S subunit (PDB ID: 3F1E) were used as the initial template for rRNA modeling. Next, the models of rRNAs (23S, 5S, 16S) were manually docked into the density map using UCSF Chimera (62). Notably, the 16S and 5S rRNAs were built by mutating the residues of *Tth* 16S and 5S sequences into *Sac* sequences. Residue insertions and deletions were performed manually in Coot following the density map. Similarly, most parts of 23S rRNA were built using the *Hma* template. For the ES and VR regions, the RNA duplex was initially generated by Coot, mutated into the *Sac* sequence and subjected to ‘real space refine’ against the density map in Coot (63). Moreover, for tRNA and mRNA modeling, the tRNA^Phe^ and mRNA template models from the *Tth* 70S–tRNA–mRNA complex structure were used as starting models by fitting them into the density map in Chimera. Lastly, new r-proteins and aEF2 were built using starting models generated from PHYRE2 (64).

## RESULTS

### Structure determination

**Figure 1.**
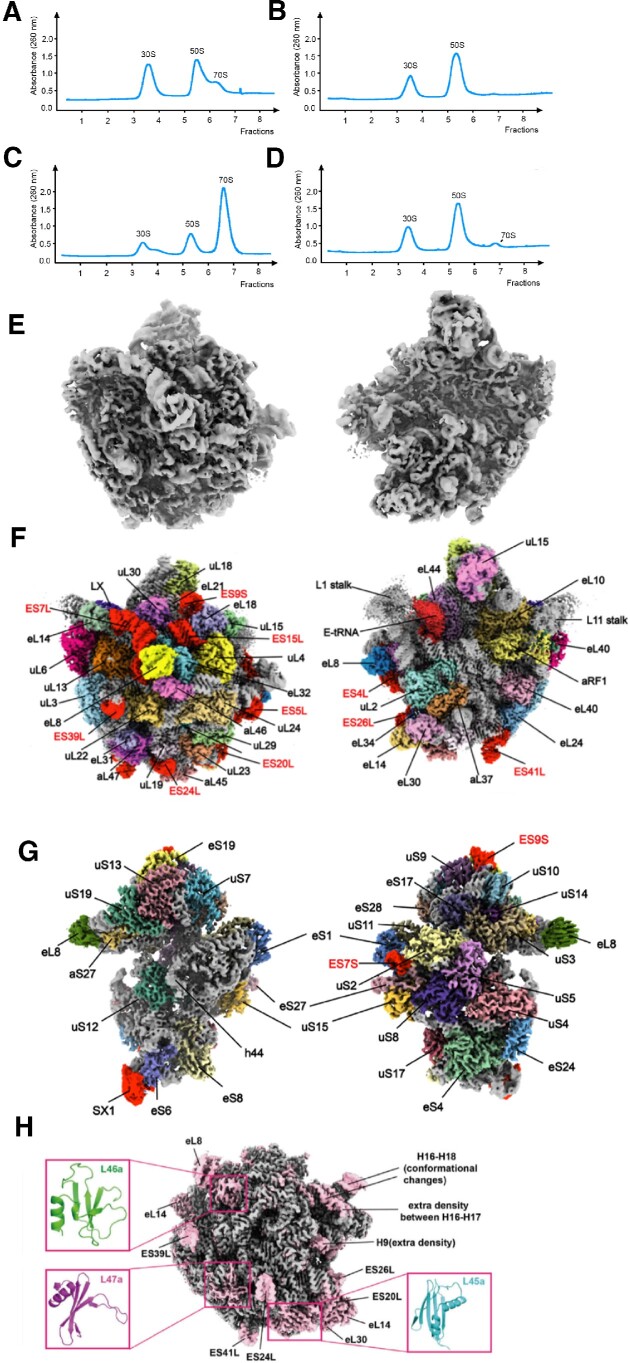
Subunit association and cryo-EM maps of *Sac* ribosome subunits. (**A**, **C**)By 10–35% sucrose gradient centrifugation, *E. coli* ribosomes with 5 mM Mg^2+^ (A) or 25 mM Mg^2+^ (C) 30S, 50S and 70S peaks were followed by absorbance at 260 nm. (**B**,**D**)The *Sac* ribosome presents as subunits under 5 mM Mg^2+^ (B) or 25 mM Mg^2+^ (D) conditions. A small peak corresponding to 70S can be detected in the 25 mM Mg^2+^ sample (D). (**E**) EM map of the 50S subunit from a cell lysis sample. (**F**) EM map of the aRF1–50S complex. Location of aRF1 and distribution of *Sac* 50S r-proteins and ribosome RNA ESs are shown. (**G**) EM density of the 30S subunit from a cell lysis sample. New r-protein labeled as SX1. h44 density is absent at this contour level. (**H**) EM density showing structural differences between the *Sac* 50S and *Hma* 50S subunits.

**Figure 2.**
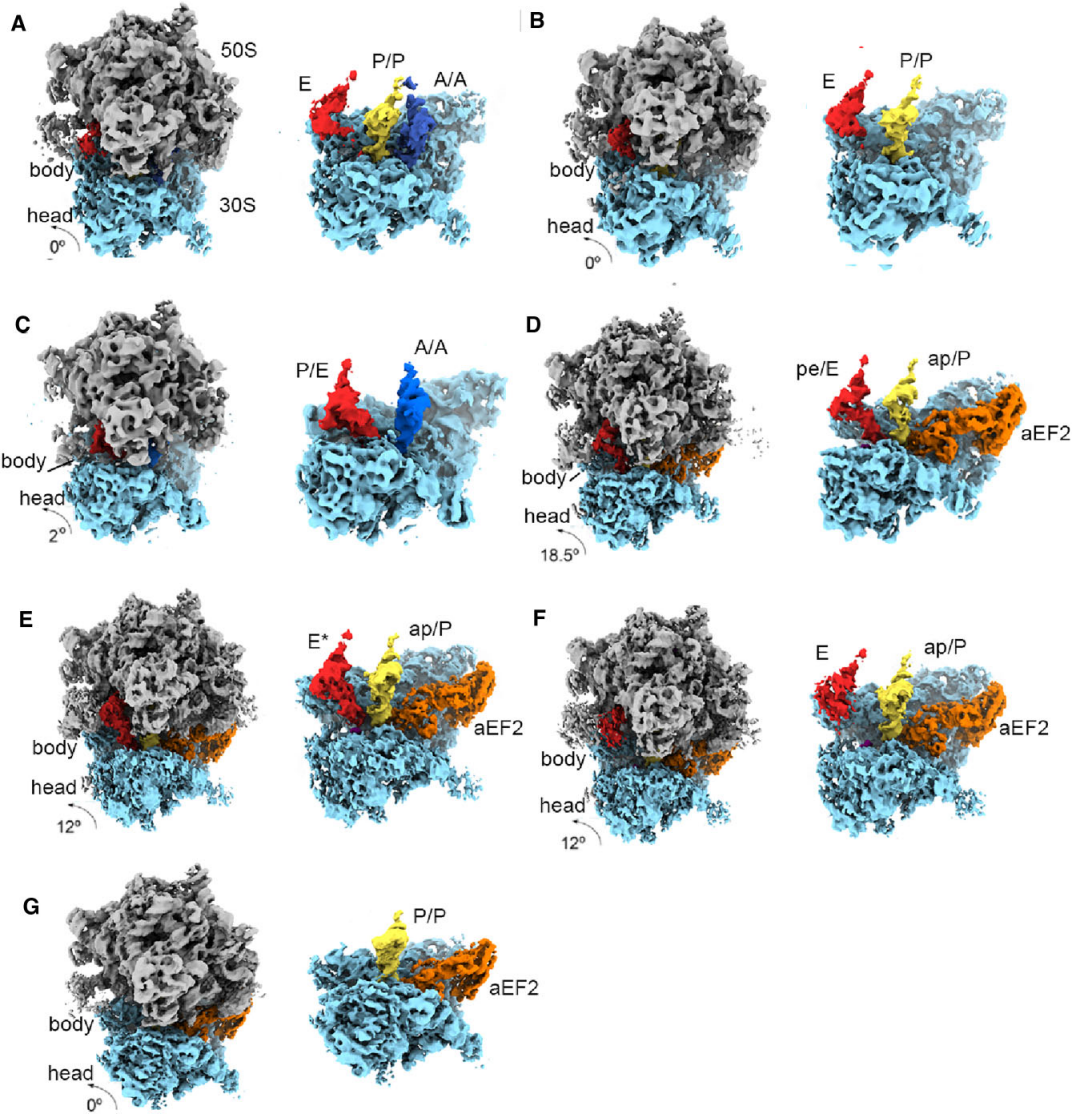
Cryo-EM maps and overall structures of *Sac* 70S ribosome complexes. (**A**) PRE state 70S ribosome bound with classical A-, P- and E-site tRNAs. (**B**) POST state 70S ribosome bound with classical P- and E-site tRNAs. (**C**) A/A tRNA and P/E hybrid state tRNA bound with 70S ribosome in a rotated state. (**D**) Chimeric ap/P and pe/E tRNAs and aEF2 bound with 70S ribosome. (**E**) ap/P and E* tRNAs and aEF2 bound with 70S ribosome with tilted 30S head domain. (**F**) ap/P and E-site tRNAs and aEF2 bound with 70S ribosome with tilted 30S head domain. (**G**) P-site tRNA and aEF2 bound with 70S ribosome in the POST state (For each panel, the 70S ribosome complexes are shown on the left, the 30S SSUs with ligands are shown on the right).

In previous studies (65,66), 70S ribosomes from *Sac* and several other sulfur-dependent thermophiles (*Desulfurococcus mobilis, Thermoproteus tenax* and *Sulfolobus solfataricus*) were weakly associated and hard purified. However, poly-phe can be synthesized using the 50S and 30S ribosomal subunits in a buffer containing spermine using the polyuridine-directed cell-free system from *S. solfataricus* (51). We first prepared crude ribosomes after cell lysis for EM analysis (sample 1, Supplementary Figure S1A and C). To obtain intact *Sac* 70S ribosomes, we incubated ribosomal subunits with their template and substrates (mRNA and Phe-tRNA^Phe^, sample 2) or translational factor aEF2 (sample 3, Supplementary Figure S1B). Moreover, in sample 4, we incubated purified *Sac* ribosome subunits with an mRNA template containing a UAA stop codon in the 3′ end and the termination (aRF1) and elongation factors (aEF1A) (Supplementary Figure S1B). Sample 1 mainly consisted of *Sac* ribosome LSU and small subunit (SSU) structures. Samples 2 and 3 contained ribosome ligands that bind the ribosome subunit interface to mediate subunit association. Furthermore, our biochemical data suggest that translation can occur when aminoacyl-tRNAs are added to samples 2 and 3. As expected, some *Sac* 70S particles were found after processing EM data from these two samples; however, no 70S particles were found in sample 4. The structures of ribosome subunits were first determined by selecting particles with large box sizes from sample 1 (Supplementary Figure S1C). Moreover, for functional complexes, we focused on 70S particles in samples 2 and 3 and applied maximum likelihood 3D classification to resolve sample heterogeneity. Classification and subsequent refinements solved structures I-A and I-B (with classical state tRNA) and structure II (with hybrid state tRNA) from sample 2. Similarly, we determined structures III–V (aEF2-engaged translocation intermediates) from sample 3. Lastly, sample 4 provided data for solving aRF1–50S complex structure (Figures 1 and 2, Supplementary Figures S1–S3 and Supplementary Table S1).

For generating an accurate 70S functional complex model, LSU and SSU structures were first built and refined based on relatively high-resolution subunits maps. The crystallographic models, such as the 50S from euryarchaeota (PDB: 1FFK), were docked into the EM map, followed by mutagenesis into *Sac* sequences. For rRNA models, molecule self-restraints generated in Coot were applied for real-space refinement. The built models were further refined in the Phenix program using base pairs, base-stacking restraints for rRNA and secondary structure restraints for r-proteins. Subsequently, the thoroughly refined subunit models were docked into 70S complex maps along with fitting tRNA, mRNA or aEF2. The initial rigid body refinement (for each ligand, 50S, 30S head and body domains) and simulated annealing refinement against EM maps enabled us to investigate local structural differences in *Sac* 70S ribosome versus bacterial and eukaryotic ribosomes.

### *Sac* ribosome association

It is unclear whether the *Sac* ribosome presents as a subunit after cell disruption or due to the purification process. The purification treatment may lead to dissociation from the 70S ribosome (67). For example, the ribosome in cells exits with high concentration but may fall apart during extended gradient centrifugation when concentration decreases substantially. Furthermore, high-concentration salt wash treatment is known to dissociate nontranslating ribosomes. Therefore, we directly performed cryo-EM analysis on the *Sac* ribosome sample after cell lysis. The 2D classification and 3D reconstruction results suggest that only ribosome subunits can be obtained upon cell lysis (Supplementary Figure S1D and E). Next, we compared the *Sac* ribosome subunit association with *E. coli* ribosomes at varying MgCl_2_ concentrations through sucrose gradient centrifugation. At low magnesium concentrations (5 mM), three absorbance peaks were observed corresponding to the 30S, 50S and 70S ribosomes in *E. coli* (Figure 1A). However, no detectable signal was observed in the region corresponding to 70S for the *Sac* ribosome (Figure 1B). Moreover, at high magnesium concentrations (25 mM), most absorbance shifted to the 70S peak for *E. coli* ribosome; however, the two major peaks for *Sac* ribosome subunits were still observed (Figure 1C and D). Furthermore, a weak absorbance for *Sac* 70S was detected at a high Mg^2+^ concentration (Figure 1D). This suggests that *Sac* ribosome subunits can associate into the 70S ribosome *in vitro*; however, the amount of 70S particles obtained was not comparable to that of *E. coli* 70S ribosome even at the same Mg^2+^ concentration. Moreover, in the ribosome samples from the 70S peak and pellet, no detectable 70S particles were obtained using EM. The few 70S may be caused by fewer subunit interface interactions in *Sac* ribosome than in *E. coli* ribosome.

### Unstable H68, H69 and h44 rRNA conformations

The LSU from sample 1 was solved at ∼5 Å resolution. We observed a helix-shaped structural element stretching from the 50S subunit association interface (Supplementary Figure S4A–G). Moreover, during model building, we noticed the lack of density for H68 and H69 of 23S rRNA based on the starting model of the *Hma* ribosome 50S subunit (Figure 1E). Additionally, H71 moved 8 Å from the original position. This suggests that the stretched density is attributed to H68 and H69. Furthermore, the density map showed a flexible upper part of H68. The molecular docking of the *Sac* 70S ribosome model on *Sac* LSU indicates that the stretched helical density clashes with the plate form of 30S SSU (Supplementary Figure S4E–G). This result suggests that LSU is incompatible with 70S formation in the current conformation. However, in the 2.7 Å aRF1–50S structure map, H68, H69 and H71 from the starting model fit very well with the density map (Figure 1F), indicating a conventional structural conformation in this complex. The cryo-EM map for *Sac* SSU distinguished most 16S rRNA base pairs. However, we noticed a relatively weak density in h44 (Figure 1G); the EM density for h44 was absent at a high contour level, but other rRNA densities were clear. At a low contour level, h44 could be traced; however, this observed density indicates the existence of other h44 conformations. In contrast, euryarchaeal (*Thermococcus celer*, EMD: 10519) or bacterial 30S (*E. coli*, EMD: 12240) EM maps from previous studies suggested a strong h44 density, indicating h44 stability in these structures (Supplementary Figure S4H–M).

The unstable characteristics of H68, H69 and h44 of ribosomal rRNA in *Sac* ribosome subunits may partly explain the difficulty of *Sac* 70S formation *in vitro*. In bacterial 70S ribosome, H68 is a component of B2a and B7a inter-subunit bridges, and H69 contacts with h44 to form bridge B2a. In contrast, the large-scale movement of H68 and H69 in the *Sac* 50S ribosome prevents the formation of these inter-subunit bridges. The mobile property of H68 was also found in *Staphylococcus aureus* ribosome when incubated at 37°C before cryo-EM (68). Notably, h44 is the longest helix of 16S rRNA spanning the 30S interface from the neck region to the spur, contributing to multiple subunit bridge interactions, including B2a, B3, B5 and B6. The mobile h44 may decrease the stability of these bridges when the 30S associates with 50S during 70S ribosome formation. We could not speculate whether H68, H69 and h44 of the rRNA in the *Sac* ribosome subunit were unstable *in vivo*. Notably, the archaeal ribosome goes through the translation initiation step in the cell before forming elongation-competent 70S (13). The initiation step involves the interplay of some initiation actors, such as aIF1 (69), aIF2 (8,9) and aIF5B (10), allowing accurate selection of initiation codon on mRNA and defining the reading frame. This contrasts with the *in vitro* 70S ribosome assembly process. The involvement of initiation factors can contribute to 70S ribosome formation.

### 50S LSU structure

In the 2.7 Å 50S subunit EM map (Supplementary Figure S5A–C), we found cryo-EM densities attributed to 10 short rRNA ESs, including ES4L, ES5L, ES7L, ES39L, ES15L, ES20L, ES24L, ES26L, ES39L and ES41L (Figure 1F and Supplementary Figure S5D–F). The size of these ESs is vastly increased in eukaryotes (5). Notably, the large rRNAs of complex organisms can increase the functionality of ribosomes. For example, in eukaryotes, the ES provides a binding hub for various nonribosomal proteins essential to ribosomal function (70). Furthermore, ESs in yeast are required for optimal growth and production of mature rRNA in ribosome biogenesis (71); however, the archaeal ES function is unexplored. Structural comparison between the *Sac* 50S ribosome and *Hma* 50S indicated that the *Sac* ribosome is structurally more complex than the *Hma* ribosome, with more abundant rRNA ESs and r-proteins (Figure 1H).

Consistent with results from 2D PAGE and mass spectroscopy, we identified the density and built *de novo* models for three *bona fide* r-proteins (aL45, aL46 and aL47; Supplementary Figure S6A–G) (66). Homolog protein search using the Dali server suggested novel folding for the three proteins (72). The r-proteins formed intensive networks with multiple rRNA helices and other r-proteins; however, the bacterial ribosome lacked components in these regions. Structural comparison with the *E. coli* ribosome indicated that aL45 and aL47 could contact the signal recognition particle in archaea (73) (Supplementary Figure S6H–J). Moreover, the proximity of aL47 and aL45 to SRP54 or the translocon suggests their coordination with these proteins during co-translational translocation. Additionally, in the SSU, we observed protein density close to the SSU spur (Supplementary Figure S6M; Supplementary Figure S6K and L shows the archaea-specific protein SX0); we termed this protein SX1. A comparison with *Tth* or yeast ribosome suggested no counterparts on their positions. Besides the above four r-proteins, two main regions contained clusters of additional r-proteins versus the bacterial ribosome. Near rRNA ES5L, three r-proteins bind to these RNA segments: eL14, eL9 and eL30. Near rRNA, H25, aL7 and eL32 bind close to this helix (Supplementary Figure S6N–Q). These r-protein distributions are similar to those of yeast ribosome r-proteins (5).

### 30S SSU structure

The previous 2.25 Å structure of *Pyrococcus abyss* 30S ribosome structure provided an atomic model of euryarchaeota SSU (10). Comparing the r-protein density of *Sac* 30S SSU with bacterial SSU showed the presence of nine additional r-proteins with new functionalities. eS24, highly conserved in archaea and eukarya, is the binding partner for ribosome recycling factor ABCE1 (74) (Supplementary Figure S6A2). Furthermore, eS28, which has no homologs in bacteria, interacted with mRNA near the E site (Supplementary Figure S6B2). We found four r-proteins in the SSU body domain and near the 16S spur: eS24, eS8, eS6 and eS4. These proteins are located above bacterial subunit bridges B8, B6 and B4. Coincidentally, *Sac* 70S lacked these three bridges (Supplementary Figure S6C2). The relationship between the presence of these proteins and the absence of the three bacterial bridges is unclear. The relative rigidity of ribosomal particles from archaea inhabiting harsh environments leads to specific molecular adjustments, ensuring efficient and accurate translation. Comparing the structure of *Sac* ribosome 30S r-proteins with that of *E. coli*, we observed that 31% more hydrophobic residues are buried in the *Sac* 30S subunit (10 265 and 7064 atoms for *Sac* and *E. coli*, respectively; Supplementary Figure S6D2–F2). These abundant hydrophobic residues are provided mostly by the nine additional r-proteins compared with the bacterial ribosome. This is consistent with prior analysis based on genomic sequences of thermophilic *Methanococcus* species (75). Furthermore, extreme thermophiles (such as *Sac*) have a considerably high ribosome subunit melting temperature than mesophilic ribosomal subunits (such as *E. coli*). This is attributed to the high ratio of hydrophobic residues in the *Sac* ribosome, which may increase conformational rigidity and compact quaternary packing to resist thermal unfolding at high incubation temperatures.

### aRF1–50S complex structure

In archaea, aRF1 forms a heterodimeric complex with aEF1A to complete the overall translation termination process in a GTP-dependent manner (76). During our termination complex formation, we first incubated aRF1 with aEF1A·GTP and added them to a ribosome–tRNA–mRNA (with UAA stop codon in the A site) complex. During the 2D classification of EM particles, we could not find *Sac* 70S particles. However, after solving the 50S LSU structure from this termination complex sample, we found the density for aRF1 and E-site tRNA (Supplementary Figure S6R).

In the 50S–aRF1 complex structure, only domains II and III were traced from the EM map; however, domain I—responsible for recognizing stop codon—was flexible, and no density was attributed to it (Supplementary Figure S6S and T). This suggests that the aRF1 domain I can be stabilized in the presence of the 30S subunit and mRNA, and no density indicates the presence of aEF1A in the 50S subunit. Moreover, aRF1 might indicate a post-termination conformation on *Sac* 50S. In the bacterial translation termination complex, class I release factor (RF1) binds to the 70S ribosome, with domain II (containing GGQ motif) swinging to the peptidyl transferase center to cleave the nascent polypeptide chain (NPC). Additionally, domain II swings 8 Å more than the RF1–70S structure (77) (Supplementary Figure S6U). aRF1 is highly homologous to eRF1, and aRF1 from archaea is active with eukaryotic ribosomes (78). We compared aRF1 in the ribosome-bound form with eRF1 in the ribosome-free or 80S ribosome-bound states (79–81). The main difference was the orientation of domain II, containing the catalytic GGQ motif. This is consistent with the conformational change of eRF1 upon recognizing the stop codon in the eukaryotic ribosome (80,81). However, in the *Sac* aRF1–50S complex, the long helix containing the GGQ motif in domain II swings 18 Å more toward the P-site tRNA CCA end, causing a steric clash with the canonical P-site tRNA (Supplementary Figure S6V–X). A structural comparison showed that the GGQ motif undergoes significant conformational changes to overlap with the backbone of residues 74 and 73 of the P-site tRNA. This finding suggests that besides releasing the NPC, aRF1 can dissociate the deacylated tRNA in the P site from the 50S ribosome after translation termination. Furthermore, inspecting domain III of aRF1 showed that it binds to the L11 stalk, and the L11 stalk RNA backbone is 7.5 Å away from that in the 80S ribosome. This difference shifts domain III 5 Å from eRF1 domain III in the 80S ribosome-bound form (Supplementary Figure S6Y and Z). This result suggests that aRF1 domain III’s binding is coupled to L11 stalk movement.

The termination mechanism in archaea differs significantly from that in bacteria: RF3 mediates RF1/RF2 recycling from the post-termination complex (82); however, archaea aRF1 acts cooperatively with aEF1A to ensure peptide release. Moreover, after aEF1A release, ABCE1 binds to the archaeal ribosome to promote aRF1 release and splits the ribosome into subunits. In the aRF1–50S structure, we did not find stretched H68 and H69 from the inter-subunit interface, suggesting that aRF1 binds with the 70S ribosome first and sticks to the 50S subunit after 70S ribosome dissociation. This is because H68 and H69 get the classical conformation in the 70S ribosome as found in our 70S complexes. Furthermore, NPCs can be found in the 70S complex structure, indicating their role in keeping the 70S ribosome intact. Therefore, NPC cleavage by aRF1 may cause 70S ribosome dissociation.

### Classical state *Sac* 70S ribosome structure

**Figure 3.**
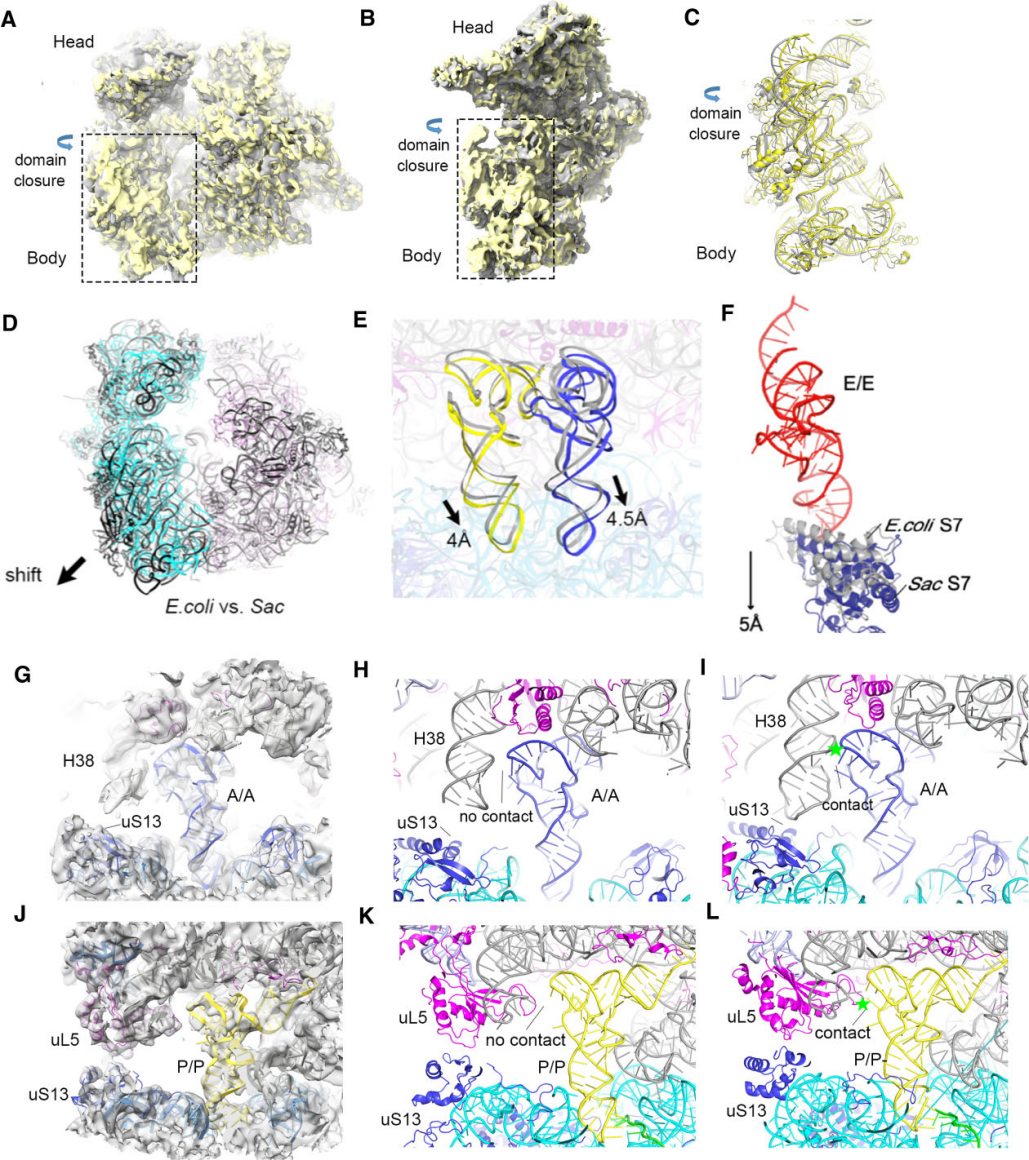
PRE (structure I-A) and POST state (structure I-B) *Sac* 70S complex structures. (**A**, **B**) Compared with the POST state, the 30S subunit body with domain closure in the PRE state can be observed. (**C**) Structural models comparing the 30S body domain in PRE and POST states. (**D**) Aligning *E. coli*’s 23S rRNA on the *Sac* ribosome showing the shift of the *Sac* 30S subunit from the subunit interface. (**E**) The shift of A- and P-site tRNAs in structure I versus that in *Tth* ribosome (based on 23S rRNA alignment). (**F**) A 5 Å movement of *Sac* uS7 (blue, caused by the 30S shift) compared with *E. coli* uS7 (gray, PDB:4V9D) eliminates interactions with E-site tRNA ASL. This leads to the absence of density for E/E tRNA ASL (based on the alignment of 16S rRNA in the 30S body domain). (**G**, **H**) H38 in structure I does not contact A-site tRNA T-arm. Additionally, H38 does not contact uS13 to form subunit bridge B1a. (**I**) P-site tRNA in *Tth* ribosome contacts H38, forming subunit bridge B1a with uS13. (**J**, **K**) uL5 in structure I does not contact P-site tRNA T-arm. (**L**) uL5 in *Tth* ribosome contacts P-site tRNA T-arm. *Tth* PRE state ribosome structure (PDB: 4V6F).

The bacterial ribosome 70S–tRNA complex has been solved up to 2 Å resolution (83). Additionally, the bacterial ribosome bound with peptidyl tRNA was solved at 2.3 Å resolution (33) or near-atomic level (34,35). However, our classical state *Sac* 70S ribosome was not solved to the atomic level and only enabled us to trace most alpha helices of r-proteins and rRNA secondary structure. This may be caused by relatively fewer *Sac* 70S particles than bacterial 70S. After calculating the classical state 70S–tRNA–mRNA complex map, we noticed that the A-site tRNA density was weaker than the P-site tRNA. This indicates sample heterogeneity regarding A-site tRNA occupancy. Next, we applied a mask on A-site tRNA and performed 3D classification (Supplementary Figure S3A) to solve structures I-A and I-B. Structure I-A contained A-, P- and E-site tRNAs, and structure I-B contained only P- and E-site tRNAs (Figure 2A and B). The major difference between structures I-A and I-B is the 30S subunit conformation. In structure I-A, we observed ∼3° SSU shoulder rotating toward the subunit interface upon A-site tRNA occupancy (Figure 3A–C). This motion of the 30S subunit brought the G496 loop of 16S rRNA (G530 in *E. coli*) and uS12 closer to the ASL of A-site tRNA. In bacteria, a key conformational change that occurs in the 30S subunit during the pre-translocation (PRE) state is the closure of the shoulder domain, maintaining the correct position of the tRNAs in the ribosome. Similarly, structure I-A represents the *Sac* ribosome in the PRE state. However, structure I-B represents the ribosome in the POST state where the 30S subunit has a regular configuration.

### Subunit interactions in *Sac* ribosomes

First, for the *Sac* 70S ribosome bound with classical state tRNA (structure I), the intact *Sac* ribosome, which sediments as 70S particles, had a diameter of ∼240 Å, significantly larger than that of the *E. coli* ribosome (∼210 Å, Supplementary Figure S6G2). Next, we compared the *Sac* 70S ribosome with bacterial 70S and eukaryotic 80S ribosomes. Based on 23S rRNA alignment, we observed large-scale movement of the 30S SSU and the 50S LSU. Furthermore, structural comparison with *E. coli* (28) 70S ribosomes showed that the *Sac* 30S ribosome body domain underwent a 3–4 Å displacement relative to the 50S subunit when viewed from the subunit interface (Figure 3D). This movement can reach 6 Å in the shoulder of the *Sac* 30S ribosome. Accordingly, *Sac* SSU shifted in the same direction as yeast or *Tth* ribosome (Supplementary Figure S6G2) (5,41). Furthermore, we compared *Sac* and euryarchaeotic ribosomes. A structural comparison indicated a very similar orientation of 30S SSU relative to 50S LSU in *P. furiosus* and *Thermococcus kodakarensis* (*Tko*) ribosomes (7,11).

During translation, the SSU and LSU of the ribosome are held together by many subunit bridges involving RNA–RNA, RNA–protein and protein–protein interactions (1). Notably, 12 bridges have been described in the *E. coli* 70S ribosome. The weak associations of the *Sac* ribosome *in vitro* prompted us to evaluate its ribosomal subunit bridges. We observed structural differences at the subunit interface compared with *E. coli* and *Tko* ribosomes, including weak interactions of subunit bridges B1a, B4, B6, B7b and B8 (Supplementary Figure S8). The reduced interactions of these bridges are caused by either displacement of the related rRNA helices or different positions of r-proteins. Notably, these differences in subunit bridge components are not caused by conformational changes upon subunit association, as their conformations are similar to those in subunit structures. Various roles have been proposed in bacteria for subunit bridges, including modulating tRNA–ribosome and factor–ribosome interactions and mediating the relative subunit movements during translocation. Moreover, mutagenesis of rRNA residues involved in *E. coli* ribosome bridges affected subunit association and increased decoding error during elongation (1,28,84). These weak subunit bridges in *Sac* ribosome may explain its reduced tRNA interactions.

### Classical state tRNA interactions in the *Sac* ribosome

EM maps and model fitting in structure I-A indicated differences in tRNA binding between the *Sac* and bacterial ribosomes. A- and P-site tRNAs shifted along with the 30S subunit in the *Sac* ribosome (Figure 3E). Moreover, the interacting ribosome components showed structural changes or shifts in their positions, resulting in considerably fewer tRNA interactions. For the A-site tRNA, H38 shifted ∼7 Å losing contact with the tRNA T-arm (Figure 3G–I). Additionally, for the P-site tRNA, the uL5 protein shifted 7 Å losing contact with P-site tRNA T-arm (Figure 3J–L). For the E-site tRNA, the density for the elbow of E tRNA was visible as this part of the tRNA contacts the L1 stalk. However, protein uS7 shifting resulted in a lack of contact with the anticodon arm and unavailable density for this part of the E tRNA (Figures 2A and 3F). We anticipated that fewer interactions between tRNAs and *Sac* ribosomes would affect tRNA translocation on the ribosome. Notably, the *Sac* ribosome naturally performs factor-free translocation (data not shown). Different tRNAs binding with the ribosome in the classical state may partly explain the underlying mechanisms.

### Structure of the archaeal ribosome bound with a hybrid state tRNA

In structure II, the ribosome featured a rotated configuration, and the P-site tRNA spontaneously moved to the E site in the LSU; however, the anticodon remained anchored on the 30S subunit, forming the P/E hybrid state (28,29,34,35). However, the A-site tRNA body maintained a classical-like (A/A) configuration, and ASL moved ∼12 Å toward the P site (Figure 2C and Supplementary Figure S7) (26,32). The movement of A/A tRNA ASL was accompanied by lateral movement of the G496 loop (G530 in *E.coli* ) and h44 of 16S rRNA, contacting ALS. Consistent with classical state tRNA bound with the *Sac* ribosome, the hybrid state tRNA-bound ribosome featured a shifted 30S compared with the bacterial ribosome in a similar state, with A/A tRNA displaced by ∼4 Å. Moreover, the P/E tRNA elbow could still contact the stalk L1 of the 50S subunit and swing into a close conformation compared with that in structure I. Additionally, the distal part of the L1 rRNA moved inward by 20 Å to contact the P/E tRNA, and the L1 protein forms a positive binding surface for tRNA interactions (Supplementary Figure S7H). These findings confirm that the L1 stalk is a mobile structure that directs tRNA movement through the ribosome during translocation, as proposed in bacteria (85).

### Structures III and V

On inspecting the cryo-EM data of sample 2, the conformational heterogeneity of the 30S head domain prompted us to perform 3D classification on this region and solve structures III–V (Figure 2D–G and Supplementary Figures S3B and S9A). Structures III and V correspond to bacterial translocation complexes recently identified by the single-molecule fluorescence method (33) or the time-resolved cryo-EM (tRNA in chimeric and POST states) (34,35) (Supplementary Figure S9B–E). Additionally, we observed the structural conservation of translocase (aEF2)-engaged translocation intermediates between archaeal and bacterial complexes (34,35). Furthermore, in structure III, the 18° SSU head rotation brought the A- and P-site tRNA ASL into ap and pe positions, and the tRNA acceptor end reached the P and E sites in the LSU (chimeric state). Lastly, in structure V, the 30S head domain swiveled back into the nonrotated conformation, and the tRNA translocated from A and P sites to P and E sites (POST state).

### New translocation intermediates

**Figure 4.**
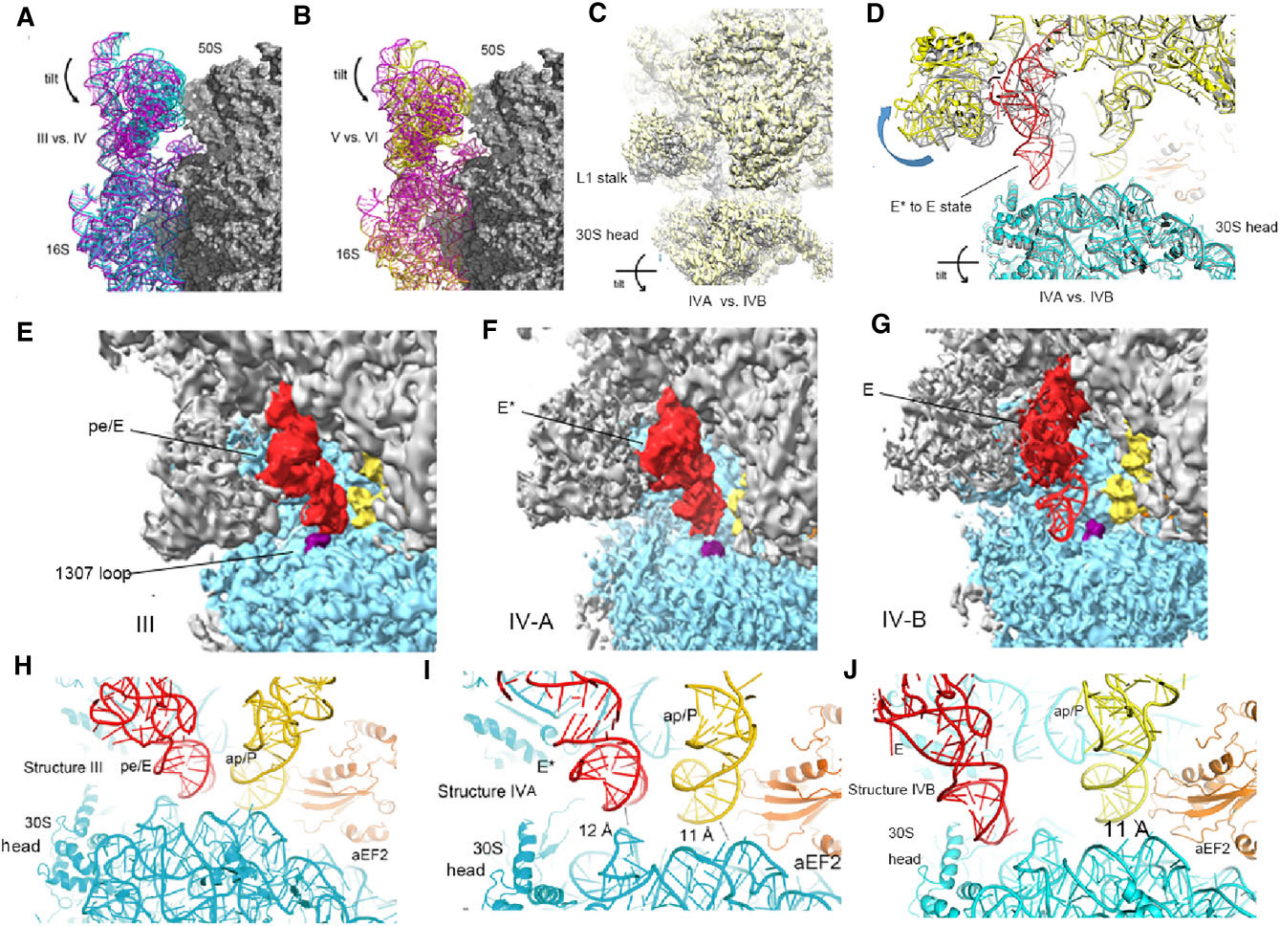
The 30S head domain tilt in structures IV-A and IV-B allows the release of translocated P-site tRNA. (**A**, **B**) Solvent-side view of the *Sac* ribosome showing the tilt of the 30S head in structure IV-A (moves outward) compared with structures III (left, chimeric state) and V (right, POST state) (based on the alignment of 16S rRNA in the 30S body domain). (**C**, **D**) Structural comparison of structures IV-A and IV-B. The main differences are as follows: in structure IV-B, L1 stalk moved outward, tRNA moved from E* state to E site and 30S head domain tilts +1°. (**E**, **F**, **G**) tRNA moves from the chimeric state (E) to the E* state (F) and E state (G), accompanied by changes in the interactions with the 30S head domain. (**H**, **I**, **J**) The 30S head (G1307 loop, corresponding to *E. coli* G1338) in structure III (chimeric state) maintains contacts with the ASL of pe/E tRNA (H); however, no such interactions exist in structures IV-A (I) and IV-B (J). The head interactions of ap/P tRNA disappear in structures IV-A and IV-B.

In structure IV, the 30S head underwent a 6° back swiveling from the fully rotated conformation (structure III), and the 30S head tilted 5° toward the solvent side (Figures 2E and F, and 4A and B). Regarding the density for intermediate P tRNA, which was not well resolved, we applied classification on this tRNA part using a mask, resulting in structures IV-A and IV-B. In structure IV-A, the P-site tRNA moved close to pe/E tRNA as in structure III. However, the tilt motion of the 30S head moved the head domain from the ASL of this tRNA. Since this tRNA was close to the E-site tRNA and did not maintain contact with P-site elements of the 30S head domain, we named it E* tRNA. Furthermore, the P-site tRNA fully moved to the E site in structure IV-B. Since the ASL part of this tRNA was not well resolved due to its flexibility and no elements of SSU directly contacted the ASL of this tRNA, we named it E-site tRNA. A structured comparison between structures IV-A and IV-B indicated that the L1 stalk in structure IV-B moved outward when the tRNA moved to the E site. Moreover, the 30S head domain in structure IV-B tilted 1° more in structure IV-B (Figure 4C and D).

In structures IV-A and IV-B, the tilt motion increased the distance between the 30S head and 50S central protuberance from 5 to 15 Å. Additionally, the dramatic tilt motion combined with the back swiveling of the 30S head enabled the release of tRNAs from the 30S head (Figure 4E–G). Notably, the A-site tRNA has been shown to move beyond the rotational movement of the 30S head during its translocation; however, the movement of the P-site tRNA is coupled to the head movement during translocation (29,33–35,39,42–50). Furthermore, in structure IV, the A-site tRNA moved to a position nearly juxtaposing P-site elements and resembling the ap/P-state tRNA. However, the distance between the 30S head and A-site tRNA ASL increased from 4.1 to 11 Å, with the body of the 30S contacting the tRNA ASL. In the chimeric state (structure III), the ASL of pe/E tRNA formed A-minor interactions with A1308 (A1339 in *E. coli*) and G1307 (G1338 in *E. coli*) of 16S rRNA in the 30S head (Figure 4H). These interactions followed the rotational movement of the 30S head during translocation. Conversely, the A-minor interactions of the 16S rRNA with P-site tRNA ASL were not observed in structures IV-A and IV-B (Figure 4I and J). Therefore, we speculated that structures IV-A and IV-B represent intermediate states between structures III (chimeric state) and V (POST state) because the head domain rotated 12° compared to 18° in structure III, and disengaged interactions of the 30S head with P-site ASL are not expected during its forward translocation.

### Structural transition from the chimeric to the POST state

Since the *Sac* 30S rRNA is structurally close to the bacterial 16S rRNA and translocase aEF2 is structurally similar to eukaryotic translocase EF2, we observed the structural differences between aEF2-engaged translocation intermediates. Here, we focused on differences in the 30S head domain, 30S body domain, ap/P tRNA and aEF2.

In structure III (chimeric state), the 30S head domain adopts a fully swiveled conformation (33–35,38,44). Next, the head undergoes a 6° back swivel from structure III to IV-A. From structure IV-A to V (POST state), the 30S head domain swiveled 12° back to the classical no-swiveled state. Moreover, the 30S body domain in structure III rotates 2°; however, structures IV-A and V show no 30S body rotation (Supplementary Figure S9F and G). Together with conformational changes on the 30S subunit, we observed gradual movements of ap/P tRNA ASL and aEF2 domain IV. The ASL of ap/P tRNA in structure III is 2.5 Å away from that in the POST state, gradually moving to an intermediate state (1 Å away) before reaching the POST state position (Supplementary Figure 2D). Moreover, we observed that domain IV of aEF2 first moves upward to contact ap/P tRNA ASL in structure III. Next, as the ASL moved into structure IV-A, domain IV of aEF2 moved 3 Å back twice until reaching the POST state as the tRNA ASL moved to the P site (Supplementary Figure S9I). Additionally, the extra insertion subdomain (different from EF-G domain IV) in domain IV of aEF2 underwent a 5 Å displacement from structure III to V (Supplementary Figure S9J). In structures III and IV-A, this subdomain contacted with 30S head rRNA; however, the interactions disappeared in structure V. Furthermore, from structure III to V, aEF2 domain displacements occurred in domain IV (the most obvious) and the other four domains. Notably, aEF2’s displacement followed the rotational movement of the 30S body (Supplementary Figure S9H). Lastly, in structures III–V, the presence of GDPNP caused conformational changes on switch loops I and II of aEF2 and stalled the translocase on the ribosome. The structural differences between these three structures suggest the coordination among the 30S subunit, aEF2 and tRNA during an EF2-catalyzed translocation.

### aEF2 engages in active conformations in structures III–V

**Figure 5.**
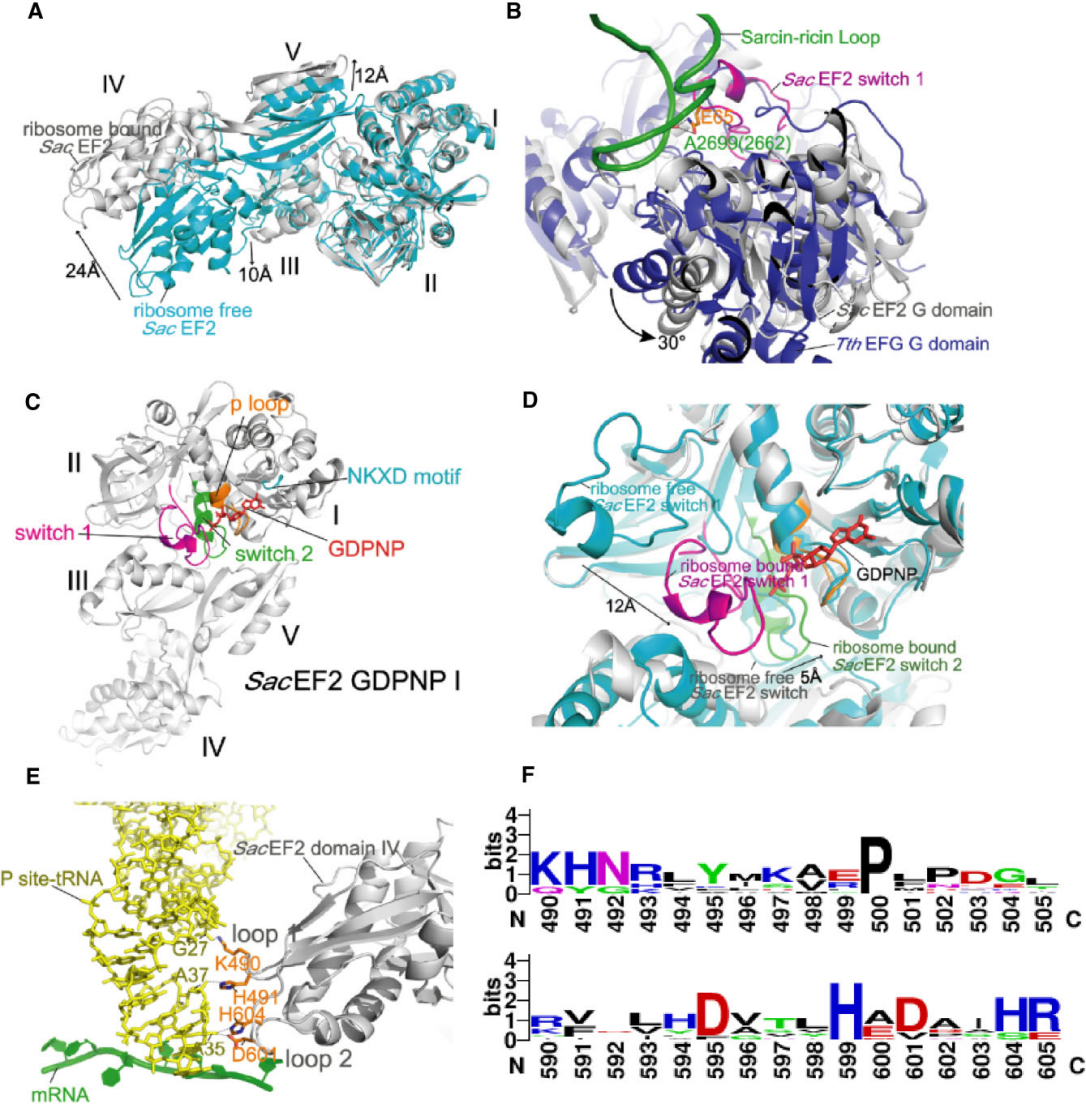
Conformations and interactions of aEF2 in the *Sac* ribosome–aEF2 complex (aEF2 from structure IV-A is shown; aEF2 conformation changes are shown in Supplementary Figure S9H). (**A**) Large-scale conformational changes of domains III, IV and IV when aEF2 switches from the ribosome-free (marked as ribosome-free state) to ribosome-bound (masked as ribosome abound) state (based on the alignment of aEF2 from residue 1–280). (**B**) A 30° rotation of aEF2 G domain relative to SRL compared with EF-G (based on the alignment of 23S rRNA of corresponding ribosome complexes). (**C**, **D**) Binding of aEF2·GDPNP on the *Sac* ribosome induces conformational changes on switch loops I and II in domain I of aEF2 (based on the alignment of aEF2 from residue 1–280). (**E**) Interactions of domain IV of aEF2 with ap/P tRNA in structure IV. (**F**) Multiple sequence alignment of residues 490–505 (loop I) and 590–605 (loop II) of aEF2 in archaea showing the conservation of loops I and II in domain IV of aEF2.

The 70S-bound aEF2 is in an extended conformation compared with its unbound X-ray form (Figure 5A). Additionally, aEF2 is bound in the subunit interface of the ribosome, where domain 1 binds to the highly conserved sarcin–ricin loop (SRL). However, domain I (the G domain) of aEF2 binds to the SRL of the archaeal ribosome in a markedly different orientation from bacterial EF-G (33–35,37,38,45) (Figure 5B). This difference may result from different subunit association patterns with the *Sac* ribosome, in which domains II and III of aEF2 moved along with the 30S ribosome. Moreover, the most significant change in the G domain of *Sac* EF2 is the conformational modification of switch loops I and II (Figure 5C and D), consistent with what happens in bacterial EF-G (29,33,34,38,44). In bacterial EF-G, switch loop I is important for its binding on the ribosome, and switch loop II is critical for ribosome-dependent GTPase activity. Notably, the restructuring of switch loop I and II regions leads to interactions with the phosphate groups of bound GDPNP, switching their conformations when aEF2 binds to the ribosome. Moreover, switch loop II rearranges to place the conserved H97 near the γ-phosphate of GDPNP and A2669 (2662). The repositioning of the catalytic histidine suggests that EF-G is active during bacterial ribosome translocation (30). Furthermore, compared with *Pyrococcus horikoshii* EF2 in the ribosome-free state, switch loop I undergoes a 12 Å displacement to contact the SRL of the 50S subunit (86), and the conserved residues E64 and Q65 of the side chain directly interact with the phosphate of G2700 (2663) and A2669 (2662) in the SRL, respectively. Therefore, the conformational changes of switch loop I fix the relative orientation of domains I, III and V through contact with domain III, stabilizing aEF2 in the extended conformation to bind with the ribosome.

### Role of aEF2 domain IV during translocation

In bacteria, the EF-G domain IV is coupled with the A-site tRNA and mRNA movement when translocating into the P site by forming interactions between conserved residues in loops I and II of domain IV and backbone phosphates of the tRNA ASL (33–35,37,45). These interactions maintain the translational reading frame (87,88). Although aEF2 is structurally similar to eukaryotic eEF2, we found similar contacts between domain IV of aEF2 and ap/P tRNA or P-site tRNA (Figure 5E). The two apical loops in domain IV engage with the minor grove of the codon–anticodon base pairs and the backbone of ap/P tRNA. Notably, K490 and H491 in loop I contacted the phosphates of G27 and A37, respectively. Furthermore, H604 and D601 in loop II interacted with the phosphate of A35 of the ap/P or P-site tRNA (Figure 5E). These interacting residues are conserved (Figure 5F). Lastly, to synchronize the tRNA movement at different translocation states, domain IV of aEF2 underwent a 6 Å displacement from the chimeric state to the POST state to preserve its contacts with tRNA (Supplementary Figures S2D and S9I).

## DISCUSSION

### 30S head tilt motion in structures IV-A and IV-B

Bacterial ribosome translocation has been well characterized (17–20,29,37,41). Recent studies using specific antibiotics (32,33), single-molecule fluorescence resonance energy transfer-guided cryo-EM (33) or time-resolved cryo-EM (34,35) uncovered several new translocation intermediates, too transient to be captured by regular cryo-EM. Moreover, molecular dynamics simulations using an all-atom structure-based model identified a new translocation intermediate with an ∼10° tilt of the 30S head (89). In our study, by investigating aEF2–*Sac* ribosome complexes, we captured two snapshots of translocation intermediate states between the chimeric and POST states. In structures IV-A and IV-B, the 30S head domain tilted 5–6° losing contact with P-site tRNA ASL. However, the ASL of E* tRNA in structure IV-A was still in contact with h23 and the side chain of Arg47 of uS11, explaining the well-resolved density for E* tRNA (Figure 4F). In structure IV-B, the E* tRNA moved to E site by 9 Å, accompanied by a 7 Å movement of the L1 stalk (Figure 4D). Moreover, in structures IV-A and IV-B, the disengaged interactions between tRNAs and the 30S head suggest that the 30S head movement can facilitate the release of tRNAs after moving on 30S. A tilting motion of the 30S head during translation is described in bacteria and eukarya (90–92). In the EF-G-catalyzed translocation complex containing transfer–messenger RNA (tmRNA), a 12° tilt of the 30S head to open bridge B1a and allow tmRNA movement during its translocation was previously described (90). Additionally, a tilt motion of the SSU head domain in the eukaryotic ribosome was reported in the structures of tRNA:mRNA (91) and Israeli acute paralysis virus internal ribosomal entry site (92) translocation complexes.

In conclusion, we investigated several ribosome complex structures from crenarchaeota, which is evolutionally close to eukarya. The structure of the aRF1–50S complex was consistent with previous findings from bacterial or eukaryotic termination complexes, in which domain II of release factors swings close to peptidyl transferase center when they bind to the ribosome (77,80,81). The structural analysis suggests that archaea aRF1 can contribute to releasing P-site tRNA during translation termination. For 70S complexes, we captured ribosomes with classical, hybrid or chimeric state tRNA bound. Additionally, the conformational changes of ribosomes upon binding the ligands were similar to those found in bacterial complexes. For example, in the hybrid tRNA-bound ribosome, we observed large-scale rotational movement of the 30S body domain and mild rotational movements of the 30S head domain. These observations were consistent with bacterial ribosome studies (25–31,33–35). Moreover, the structures of archaeal 70S ribosome complexes support the idea that the mechanisms of the translation elongation process are conserved in different domains of life (17–22). One difference we found for the 70S ribosome is its weakly associated property. This leads to some differences in tRNA binding versus bacterial ribosome. How these differences are related to archaeal translation in extreme conditions needs further studies.

## Supplementary Material

gkad661_supplemental_file

## Data Availability

The cryo-EM maps for 30S SSU, 50S LSU, 50S–aRF1, and structures I-A, I-B, II, III, IV-A, IV-B and V have been deposited with the accession numbers EMD-34862 (30S), EMD-34861 (50S), EMD-34860 (50S–aRF1), EMD-34863 (structure I-A), EMD-34864 (structure I-B), EMD-34870 (structure II), EMD-34868 (structure III), EMD-34866 (structure IV-A), EMD-34867 (structure IV-B) and EMD-34869 (structure V), respectively. The atomic models of the above structures have been deposited with the accession numbers 8HKX (30S), 8HKV (50S), 8HKU (50S–aRF1), 8HKY (structure I-A), 8HKZ (structure I-B), 8HL5 (structure II), 8HL3 (structure III), 8HL1 (structure IV-A), 8HL2 (structure IV-B) and 8HL4 (structure V), respectively.
