# Supplementary material for: Cryo-electron microscopy structure and translocation mechanism of the crenarchaeal ribosome
Source: Nucleic Acids Res. 2023 Aug 22;51(17):8909–24. doi: 10.1093/nar/gkad661 (PMC10516650; doi:10.1093/nar/gkad661)
Supplement: gkad661_supplemental_file [file gkad661_supplemental_file.docx]

**Supplementary Materials**

**Insights into the molecular mechanisms of factor-free translocation**

We sought to investigate the possible molecular mechanisms involved in factor-free translation based on *Sac* ribosome structure. We focused on the structural features that potentially contribute for spontaneous ribosome translocation. First, studies have proposed that *E. coli* ribosomal proteins uS12/uS13 function as control elements for tRNA:mRNA translocation (1). We observed considerably shifted S12/S13 position in our structures (Supplementary Figure 7). S12 in bacterial ribosomes was found to extensively contact the D-arm of A/T state tRNA in the preaccommodation state(2). Moreover, S12 was found to be very close to the codon–anticodon helix in at A-site (3). Interactions between A-site tRNA and S12 must change as the ribosome progresses through the translation cycle. In our *Sac* 70S ribosome structures, we observed that S12 shifts 5 Å away from its position in the bacterial ribosome. Thus, S12 does not form direct interactions with either A/A or A/T state tRNA (Supplementary Fig. 8K-N). In the bacterial ribosome, S13 is located in the head domain of the 30S subunit (4). The N terminus of S13 makes direct contact with the central protuberance of the large ribosomal subunit through subunit bridges B1a and B1b (5). By contrast, neither bridge B1a or B1b in the *Sac* ribosome were found with strong interactions in classical state 70S complexes (Supplementary Figure 7). Because bridge B1a is a translocation attenuator (6,7) and both B1a and B1b in bacteria constrain the movement of the 30S head domain during translocation, the absence of B1a and B1b bridges suggests that the constrain for 30S head movement is eliminated during tRNA translocation in *Sac.* In addition to bridge B1a and B1b, the elimination of bacterial ribosome subunit bridges B4 and B8 in *Sac* is important for spontaneous translocation. Mutations that disrupt bridges B4 and B8 in the *E. coli* ribosome increased the maximal rate of forward translocation (8). Thus, breaking these bridges can lower the energy barrier of ribosome translocation. Finally, the absence of constraints for the A-site and P-site tRNA elbow movement and E-site tRNA ASL release by uS7 are observed in the *Sac* ribosome. These absent tRNA interactions may contribute to factor-free translocation.

**Detailed image processing**

For *Sac* ribosomal subunits, 1,522,987 particles were selected from sample 1 dataset after particle picking from crYOLO. For the picking model, the one from cryo-EM images which was trained on low-pass filtered images was used. The output .star files were combined and was used for particle extraction after CTF estimation with CTFFIND-4.1. The extracted particles were imported into cryoSPARC. Down sample particles were applied by setting fourier crop to box size 180 pix. After 2D classification (setting number of 2D classes to be 200), 30S and 50S were selected separately and were used for Ab-Initio reconstruction and homogeneous refinement. To get rid of “junk” particles, four separate Ab-Initio jobs were run to identify particles that gave promising subunit EM maps. For 50S LSU structures, using un-binned particles lead to poor density on many regions. We used the 2X binned particles for refinement and got the 4.94 Å map. aRF1-50S structure was obtained in similar way. Non-uniform refinement was used for improving resolution with 0.1-0.2 Å

For the 70S ribosome·tRNA^Phe^·mRNA complex dataset, 1,850,437 particles were picked from 6153 micrographs by crYOLO, followed by estimation of CTF parameters and particle extraction. All particles were then imported into cryoSPARC for multiple rounds of 2-D classification to remove subunits particles and bad particles. 15,829 70S particles were picked from this dataset for a single class *Ab-initio* reconstruction and homogeneous refinement. The initial model was imported to RELION and was low-pass filtered to 60 Å for using as a reference of 3D refinement. The optimizer.star file generated from 3D refinement were used for subtraction of signal outside of 30S subunit. 3D classification with six classes was then performed. Two major classes were identified with substantially different 30S orientation. For one class that containing density for the A-site, P-site, and E-site tRNA were selected for further subsorting since the A-site tRNA was not well resolved. A mask generated from A-site tRNA pdb model was used as focus mask for classification on cryoSPAC. From this step, structure I-A and I-B were solved separately. Another class contains the density for A/A tRNA and hybrid P/E tRNA with significant 30S body rotation. After homogeneous refinement, the final resolution was estimated to 5.72 Å for the rotated 70S ribosome containing P/E hybrid state tRNA (structure II), 4.45 Å for the PRE state 70S ribosome containing A/A, P/P and E/E tRNAs (structure I-A), and 4.78 Å for POST state 70S ribosome containing P/P and E/E tRNAs (structure I-B). Supplementary Fig.2A summarized data processing details.

For the 70S ribosome·tRNA^Phe^·mRNA·aEF2·GDPNP complex dataset, a total of 1463220 particles were picked from 5091 micrographs followed by estimation of CTF parameters and particle extraction. We sorted out 18,527 70S particles from 1,463,220 particles for one class *Ab-initio* reconstruction. Next, the 70S density map was generated from *Ab-initio* reconstruction and homogeneous refinement. The particles from cryoSPAC were imported into RELION for 3D refinement and particle subtraction by using 30S head as a mask. 3D classification was performed with six classes, T=200.Three classes were identified and represented three distinct conformations of the 30S head domain. The corresponding particles from these three classes were separately used for 3D refinement, yielding three structures of *Sac* ribosome·aEF2 complexes (structure III, IV and V). We noticed that the E* tRNA in structure IV was not well resolved. This prompt us to perform further sorting of particles from this class by using E* tRNA mask. This led to solving structure IV-A and IV-B. The final resolution of these four structures are 4.8 Å (structure III), 3.93 Å (structure IV-A), 4.1 Å (structure IV-B) and 4.62 Å(structure V) respectively. Supplementary Figure 3B summarized data processing details.

**Model building and refinement**

The crystal structure of the *Haloarcula marismortui* (*Hma*) 50S large subunit (PDB ID:1FFK) and the crystal structure of *Tth* 30S subunit (PDB ID: 3F1E) were used as the initial template for rRNA modeling. The models of rRNAs (23S, 5S, 16S) were docked into density map manually using UCSF Chimera(9). The 16S and 5S rRNA was built by mutating the residues of *Tth* 16S and 5S sequence into *Sac* sequences. The residues insertion and deletions were performed manually in Coot to follow the density map. Similarly, most parts of 23S rRNA were built in similar strategy by using *Hma* template. For the ES and VR regions, the RNA duplex was initially generated by Coot, and was further mutated into *Sac* sequence, and was subjected to “real space refine” against the density map in Coot(10). For fitting RNA duplex, restrains generated from ProSMART was used together with real space refinement. For modeling of tRNA and mRNA, the tRNA^Phe^ and mRNA template models from a *Tth* 70S-tRNA-mRNA complex structure were used as starting model by fitting into the density map in Chimera. For the mRNA, the density map only allows to trace the bases in the A, P, E-site even we use a 29 nucleotides long mRNA in the complex preparation. It indicates the flexibility of mRNA in the downstream.

For modeling of the three new r-proteins (aL45, aL46, aL47), the sequences of these three proteins were subjected to 2D and 3D structure prediction, using JPred(11) and PHYRE2(12) respectively. Initial fitting of these three r-proteins was guided by high agreement of secondary structural features between the predicted 3D models and the density map. Taken aL45 for example, the predicted model was first switched into a poly-analine model by using CHAINSAW in the CCP4 suite(13) and fit into the density map in Coot. Subsequently, the five beta strands and two alph-helix were adjusted into the density manually with the information from secondary structural prediction. Several regions of the beta stands and many loop regions did not fit the map well and were adjusted accordingly. The sequence assignments were aided by well resolved bulky residues such as Phe, Tyr. Trp. The side chains were adjusted by using “real space refine” and “rotamers refine” in Coot^1^. aL46 and aL47 were built in similar process. For modeling of *Sac* aEF2, the crystal structure of *Pho* EF2 bound with GDPNP was used as template and was subjected to SWISS-MODEL(14) for protein structure homology-modeling (sequence identity 51%). Next, the output model was first fit into the 3.9 Å density map (structure IV-A) in Chimera based on the orientation of domain I and domain II. The positions of domain III, IV, and V of *Sac* aEF2 were adjusted manually in Coot and Chimera. For the linker loops which connect each domain, manual fitting was performed in Coot according to the density map by using “regularize refine.” Because each domains’ position of aEF2 is significantly different from that in ribosome-free state, many side chains and several main chain of aEF2 was adjusted according to the density map. GDPNP molecule density is very clear and straightforward for rigid body fitting. The conformational changes of switch loop I and II do not allow rigid fitting of corresponding model from the predicted model. All residues in these two regions were mutated into poly-alanine and adjusted manually. Because the density merging feature by decreasing the contour level of the map for the key residues that contact SRL, tRNA ASL and the head domain of 30S, we carefully adjust each residues by manual fitting with “rotamers refine” and “chi angles refine” in Coot. For modeling of ribosomal proteins except aL45, aL46, and aL47, the r-protein models from crystal structures of *Haloarcula marismortui* 50S large subunit, *Tth* 30S subunit and *Saccharomyces cerevisia* were used as starting model for homology-modeling in SWISS-MODEL. After generating all these models, they were separately aligned in Pymol to their binding positions based on the above crystal structure models. Next, the models of r-proteins, tRNA, mRNA, 23S rRNA, 5S rRNA, 16S rRNA, and aEF2 were merged into a single PDB file and be fitted into the cryo-EM density map by using Chimera.

The atomic models of 50S subunit, 30S subunit and two 70S complexes were refined against the density map in PHENIX. Taken structure IV for example, two rounds of rigid body was performed first. The fist round was run by defining 50S, 30S body, ap/P, E* tRNA and aEF2 as rigid body groups. The second round was run by using separate r-proteins and rRNA chains as rigid body groups. Subsequently, one round of simulated annealing refinement (slow cooling from 2500 T) was run to solve local structural changes. After that, real space refinement (phenix_real_space_refine) was run with secondary structure and geometry constrains applied. After this refinement, manual adjustments of tRNA, mRNA and aEF2 was done in Coot and was following by another round of real space refine. The statistics of the refined model were generated from phenix refinement log files. The cartoon images in main figs or extended data figs were generated with The PyMOL Molecular Graphics System, Version 1.2,


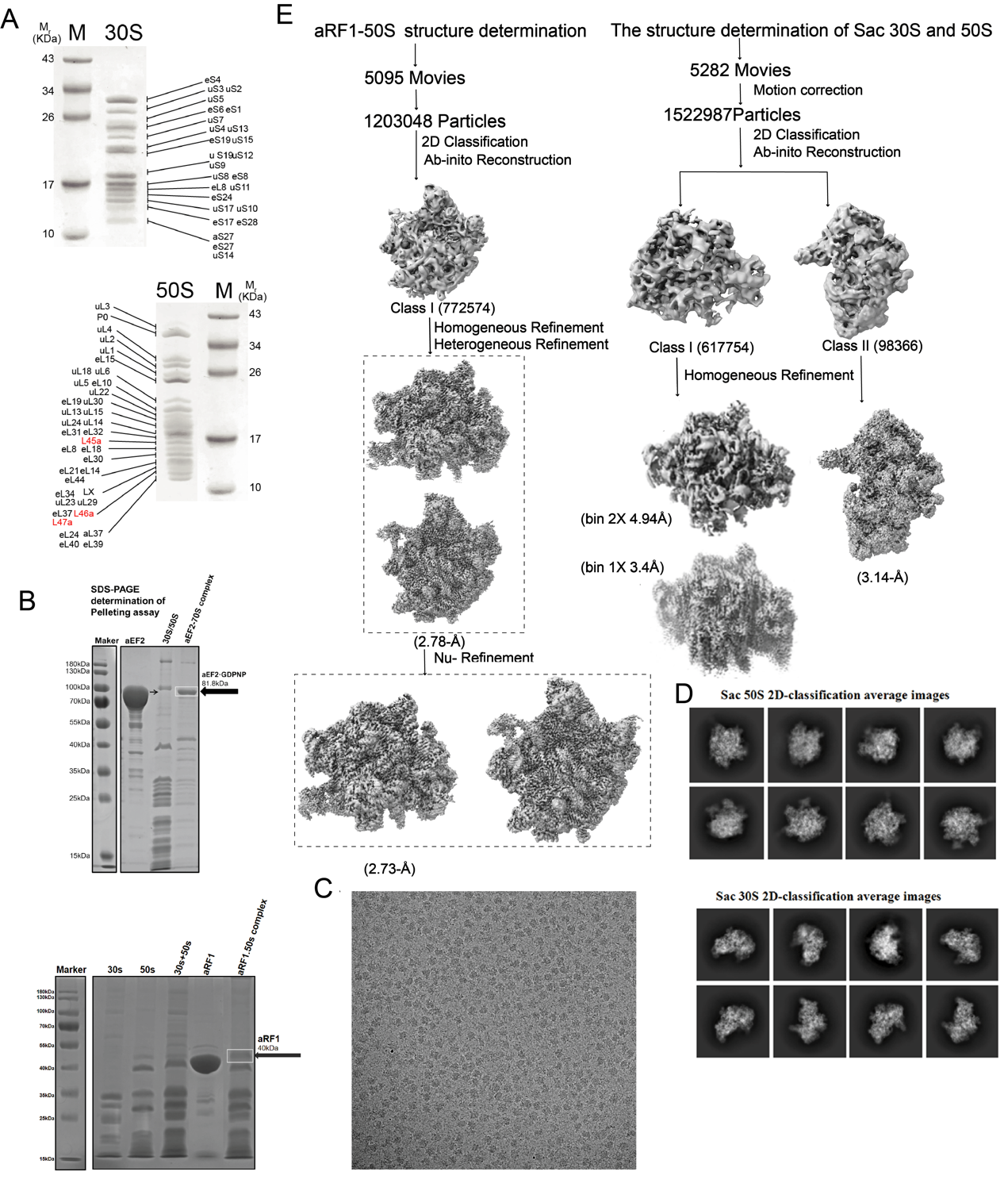


**Supplementary Figure 1. Analysis of *Sac* ribosome and functional complex, scheme for the cryo-EM image processing of *Sac* ribosome subunits and comparisons of five Sac ribosomal functional complexes**

(**A**) SDS-PAGE identified all ribosomal proteins from the two subunits. The new r-proteins, aL45, aL46, and aL47 are shown in red.

(**B**) The pelleting results indicate the binding of aEF2 or aRF1 to the *Sac* ribosome.

**(C)** Data collection images of crude ribosome after cell lysis (after motion correction).

(**D**) Representative 2D class average images of subunits particles.

(**E**) Procedures for cryo-EM data processing and structure determination of SSU and LSU and aRF1-50S datasets. For LSU structure determination, 2X binned particles gave the best resolution. Thus, the final map was generated base on 2X binned particles. (please refer to Methods for details).


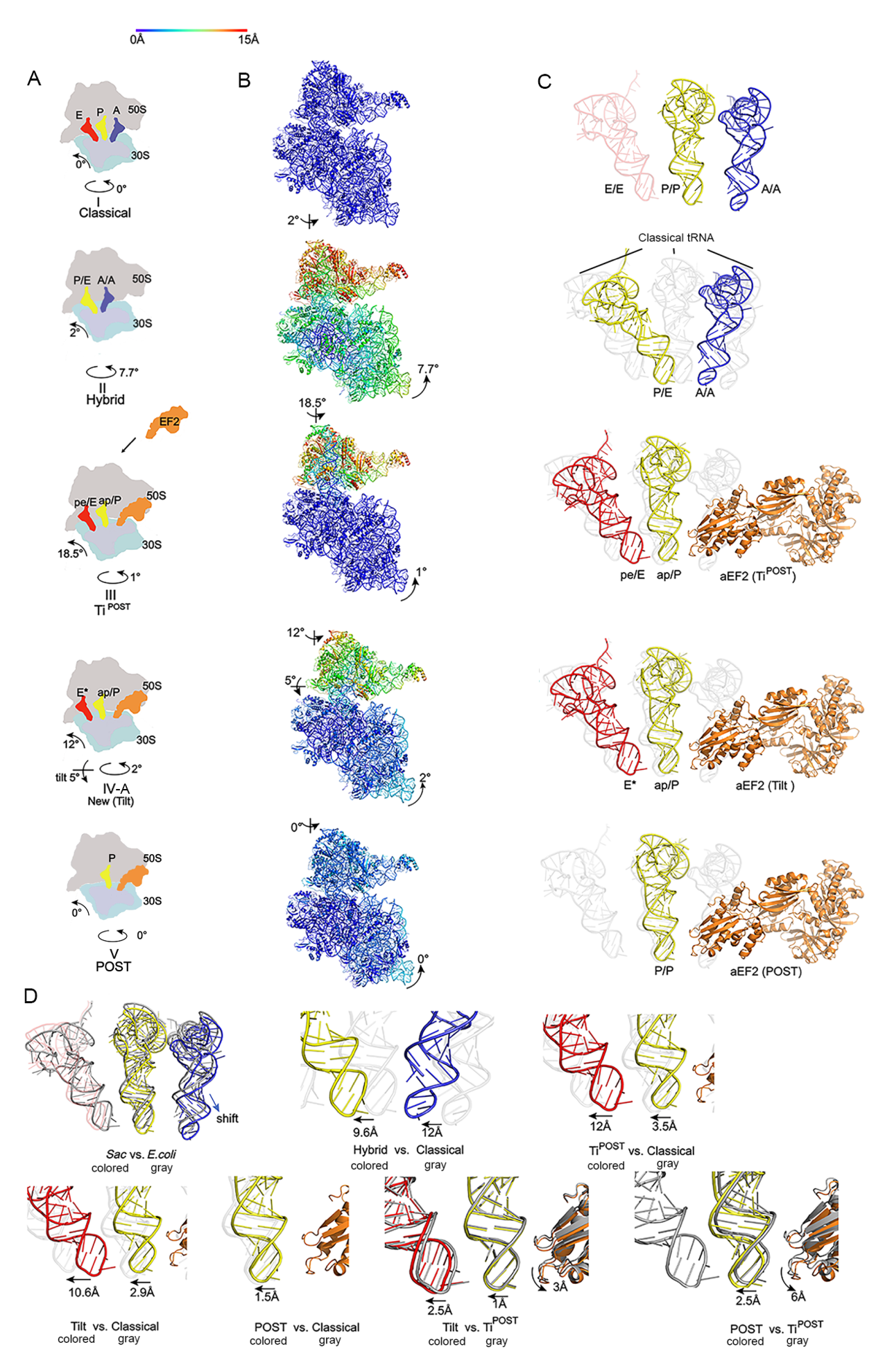


**Supplementary Figure 2: Structural comparison of 30S SSU, tRNAs and aEF2 in different states.**

(**A**) Schematic of translocation reaction coordinate in archaea depicting SSU rotation (blue) with respect to the LSU (gray) and SSU head-swivel (purple). tRNAs are colored on a gradient from the A (blue) to P (yellow) to E (red) sites. aEF2 is colored orange.

(**B**) The conformational changes of SSU accompanying each sequential translocation step, viewed from inside the inter-subunit space, colored by r.s.m.d. at each SSU residue for each transition (structure I-B was used as reference model for the color scheme).

(**C**) tRNA (A/A: blue, P/P or P/E or ap/P: yellow, pe/E or E* or E/E: red) and aEF2 (orange) positions relative of classical state tRNAs during each step of translocation.

(**D)** Alignment of LSU core showing (1) shift of *Sac* tRNAs (colored) relative to *E.coli* tRNAs (gray). (2) distances of tRNA ASLs in hybrid state (colored) relative to classical state (gray). (3) distances of tRNA ASLs in chimeric hybrid state (colored) relative to classical state (gray). (4) distance of tRNA ASLs in the new tilt state relative (colored) to classical state (gray). (5) Distance of tRNA ASLs in the POST state (colored) relative to classical state (gray). (6) Conformational difference of aEF2 domain 4 (orange) and position of tRNAs (colored) in the new tilt state relative to that in Ti^POST^ state (gray). (7) Conformational difference of aEF2 domain 4 (colored) and position of tRNAs in the hybrid state relative to that in POST state (gray).


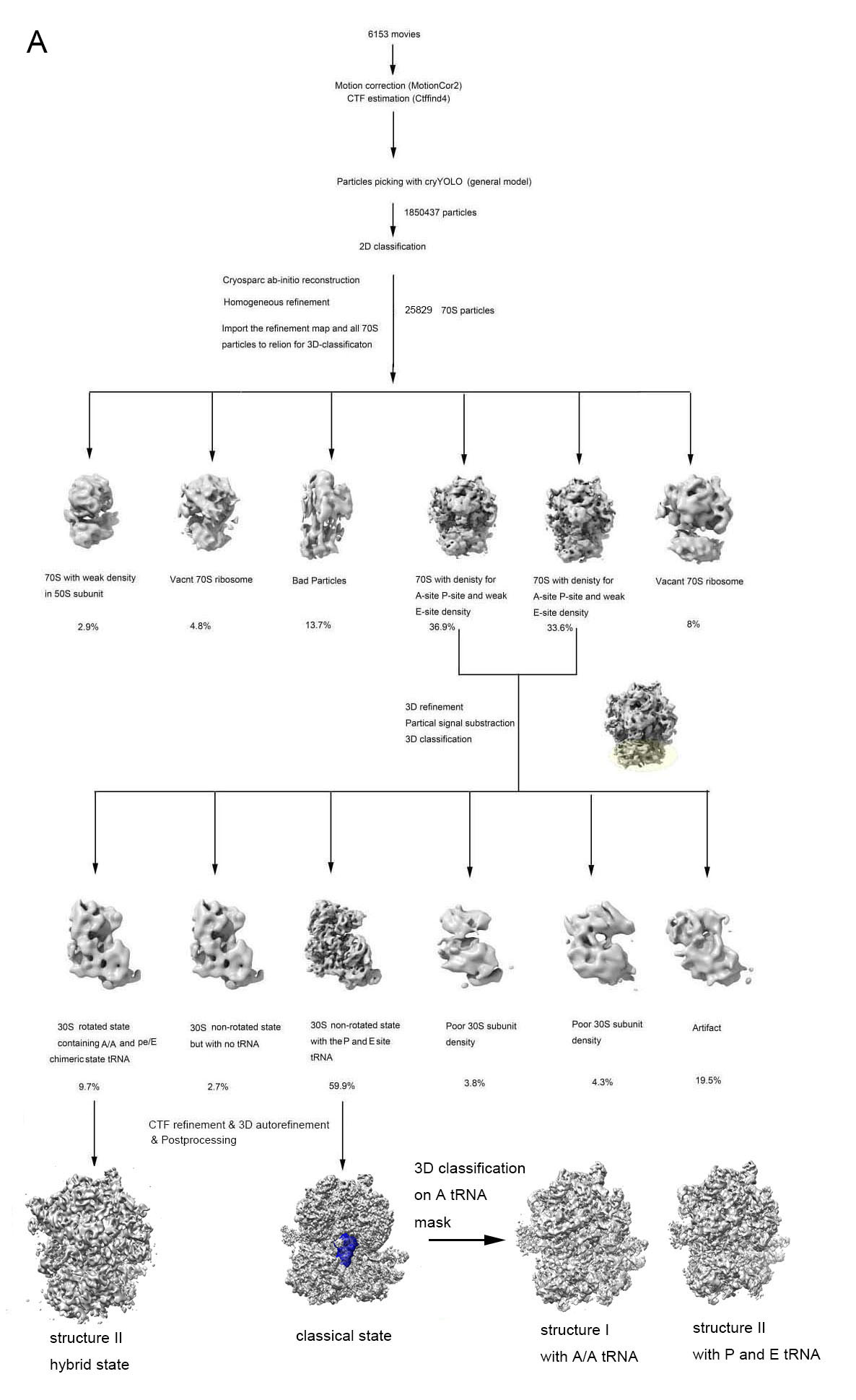


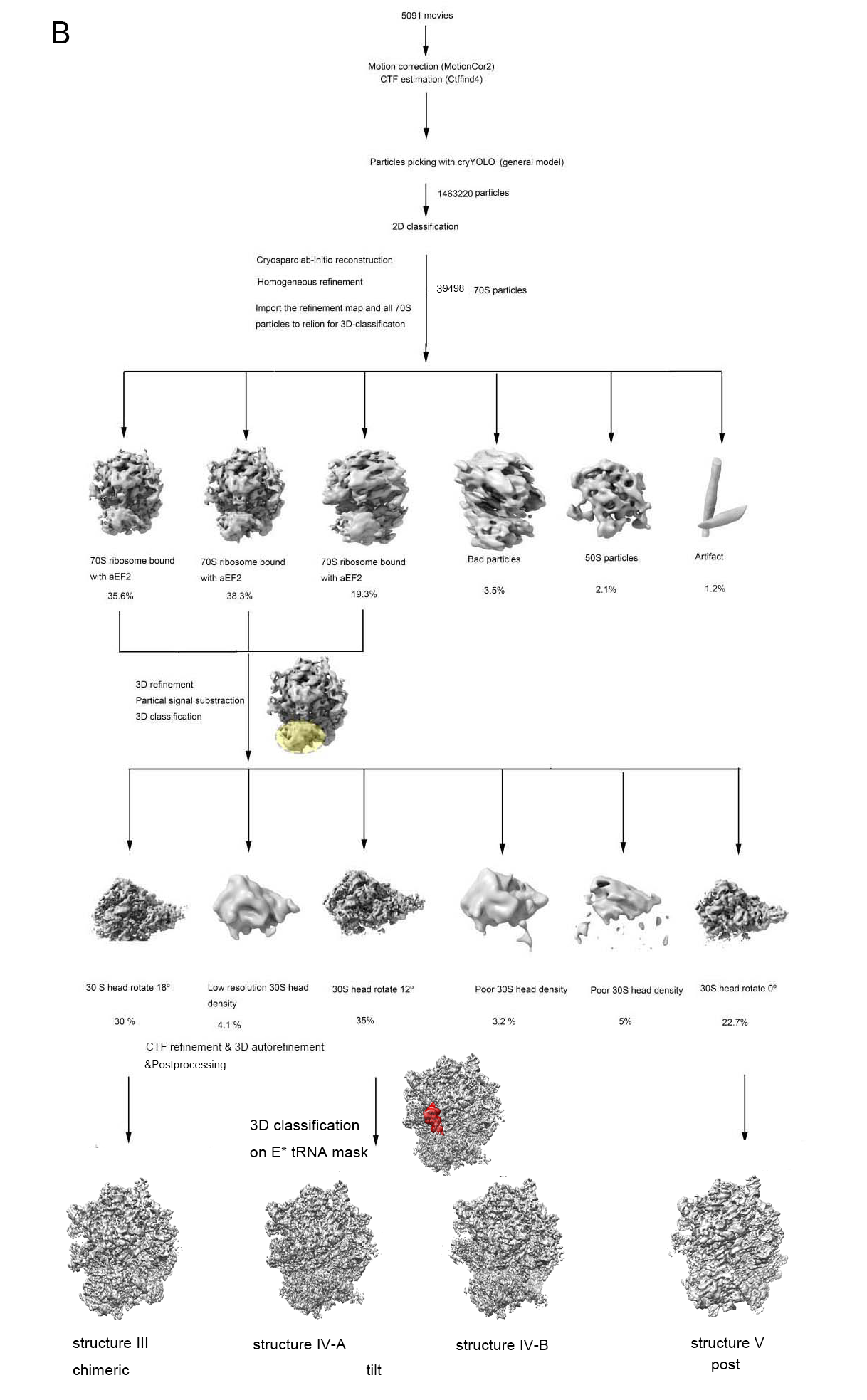


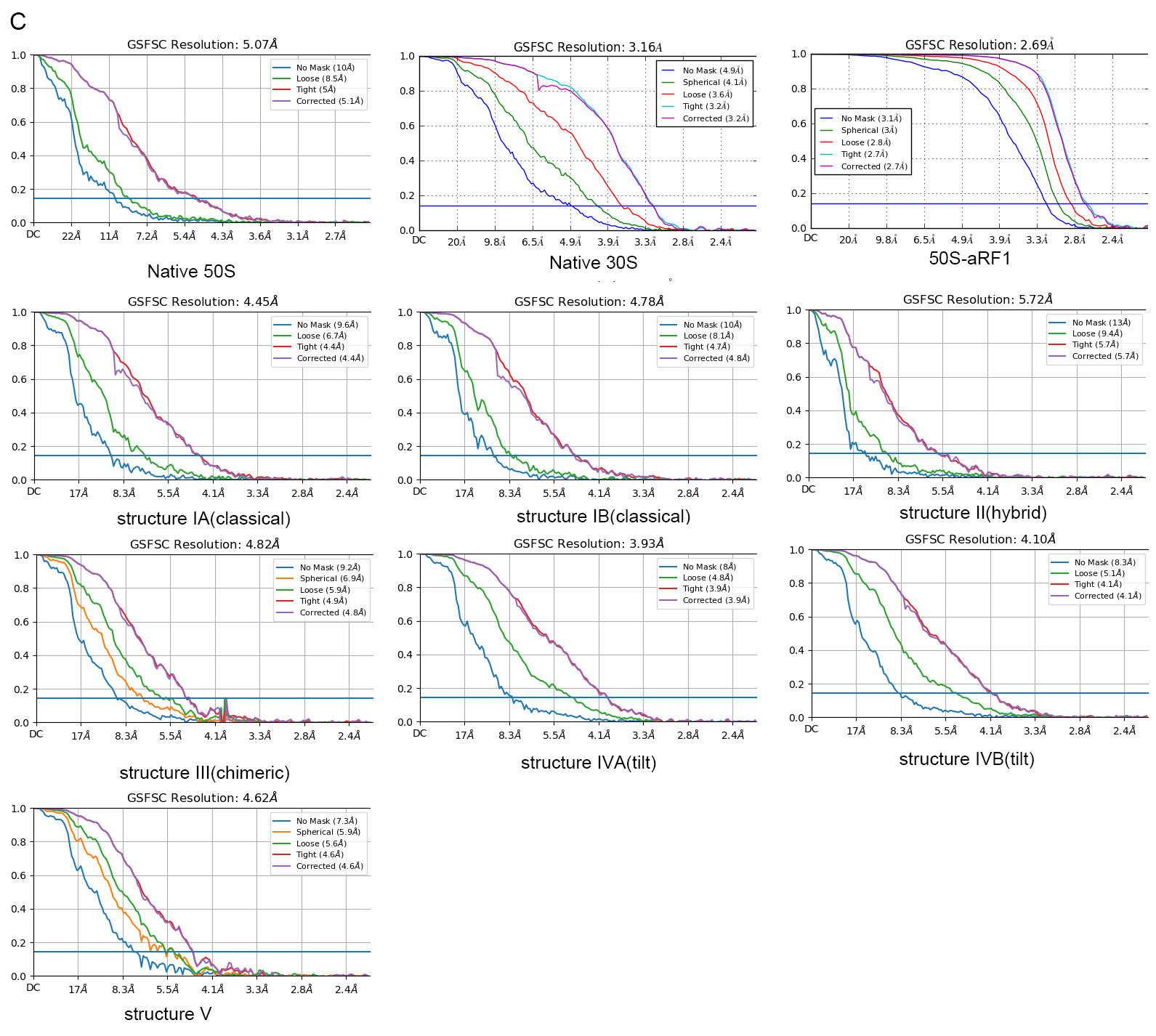


**Supplementary Figure 3. Cryo-EM data processing of particles from sample 2 (A), sample 3 (B), FSC curves are shown in (C).**

(**A**) Initially, the 70S particles were picked up based on the 2D selection from the 2D classification results. The 70S particles data were subjected to two rounds of 3D classification in RELION 3.1. The first round excluded bad particles and vacant 70S particles. The second round classification was performed using a mask of the 30S subunit (after particle subtraction), which lead to the identification of two major classes with different 30S rotation degrees relative to the 50S subunit. The particle reconstructions and refinements from these two classes provided the structures I and II (50S, gray; 30S, cyan; A-site tRNA, blue; P-site tRNA, yellow; E-site or P/E tRNA, red). Due to the low occupancy found in structure I map, a mask on A-site tRNA was used for further particle sorting and lead to solving structure I-A and I-B.

**(B)** After 2D particle classification, the 70S ones were selected and subjected to two rounds of 3D classification in RELION 3.1. In the first round, bad particles and 50S contaminations were removed. In the second round, classification was performed based on a mask around the 30S head domain (after particles subtraction). Three major classes were identified (different rotation degrees of 30S head relative to 30S body domain) and particles reconstruction and refinement lead to structures III, IV, and V (50S, gray; 30S, cyan; ap/P or P-site tRNA, yellow; pe/E tRNA, red;). EM map of E* tRNA indicates particles heterogeneity. A mask on E* tRNA was used for further classification and lead to solving structures IV-A and IV-B.

**(C)** FSC curves generated by CryoSPARC after homogeneous refinement of each structure by using particles after particle sorting.

(Explanation of FSC curve by CryoSPARC:

No Mask: This is the raw FSC calculated between two independent half-maps reconstructed from the data. There is no masking applied, so both the structure and solvent are included in this FSC

Spherical: This is the FSC calculated after applying a soft spherical mask to both half maps. The outer radius of the soft sphere is equal to half the volume box-size. The inner radius is 85 percent of the outer radius. Between inner and outer radii, a soft cosine edge transitions from a mask value of one to a value of zero.

Loose: This is the FSC calculated after applying a soft solvent mask to both half maps. The loose mask is calculated as follows. First, the density map is thresholded at 50% of the maximum density value. The resulting volume is dilated to create a soft mask. Voxels in the mask that are within 25 angstroms of the thresholded region receive a mask value of 1.0. Voxels between 25 and 40 angstroms fall off with a soft cosine edge, and voxels outside 40 angstroms receive a value of 0.0.

Tight: This is the same as the loose mask, except the dilation distances are 6 angstroms for the value 1.0 distance and 12 angstroms for the value 0.0 distance.

Corrected: This is the FSC curve calculated using the tight mask with correction by noise substitution. The two half maps have their phases randomized beyond a certain resolution, then the tight mask is applied to both, and an FSC is calculated. This FSC is used along with the original FSC before phase randomization to compute the corrected FSC. This accounts for correlation effects induced by masking. The resolution at which phase randomization begins is the resolution at which the no-mask FSC drops below the FSC = 0.143 criterion.)


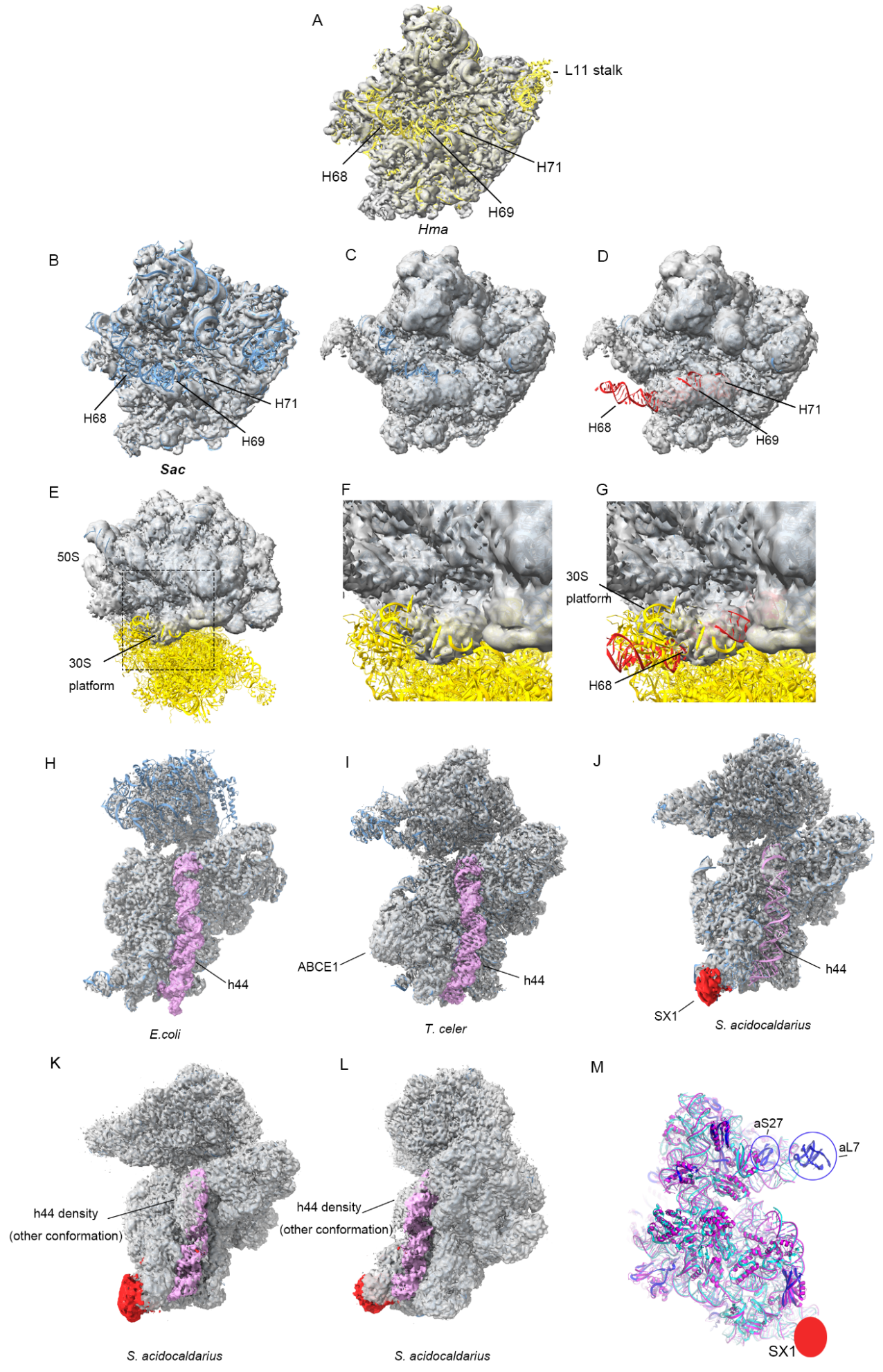


**Supplementary Figure 4. Unstable H68, H69 and h44 of 23S rRNA**

**(A)** Fitting of *Hma* 50S model into *Sac* 50S map (PDB ID:1FFK). There is no density attributed to H68 and H69. H71 is also outside of the map**.**

**(B)** Fitting of 50S model into 50S ribosome map with *Sac* 50S refined from 2.7 Å map (aRF1-50S complex).

**(C)** At low contour level, a helical shaped density was observed to stretch out from subunit interface.

**(D)** Adjusting the model of H68 and H69 against 50S subunit map indicates conformational changes of these two helices.

**(E)** Docking *Sac* 70S model (from structure I-B) into 50S subunit map suggests clash of stretched density with 30S subunit.

**(F-G)** Close-up view showing that stretched H68 density clash with 30S platform.

**(H)** Cryo-EM map of *E.coli* ribosome 30S subunit structure (EMDB: EMD:12240)

**(I)** Cryo-EM map of *Thermococcus celer* 30S-ABCE1 structure (*T.celer*, EMDB: 10519)

**(J)** Cryo-EM map of Sac ribosome 30S subunit structure. h44 density at high contour level is very weak, indication structural instability.

**(K-L)** At low contour level, h44 density become visible. The extra density outside conventional h44 model suggest the existence of other conformation.

**(M)** Structure comparison of *Sac* 30S with *Thermococcus kodakarensis* (*Tko*, PDB:6SKF) 30S subunit. *Sac* contains three more r-protein: eL27, aL7 and SX1.


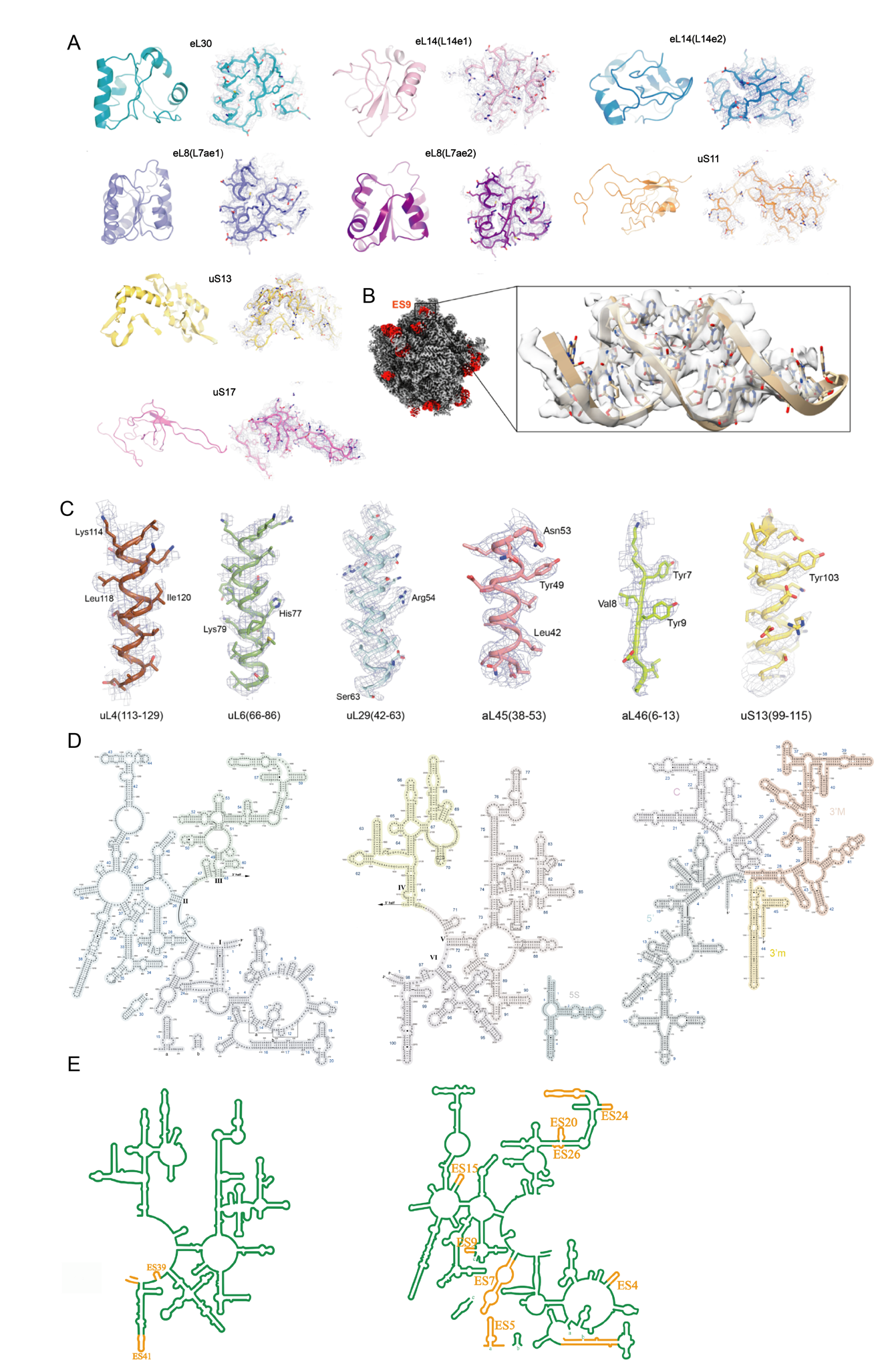


**
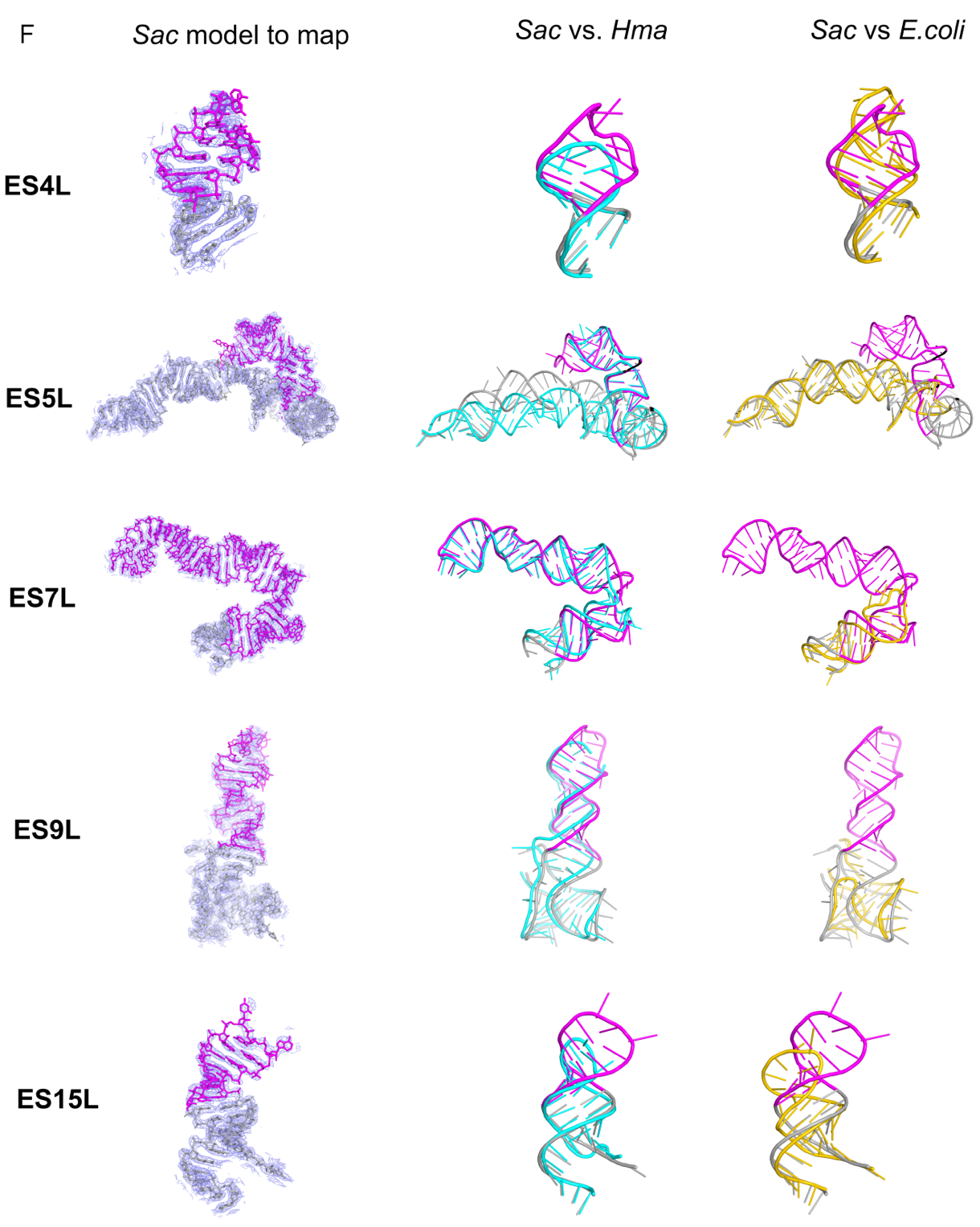
**

**
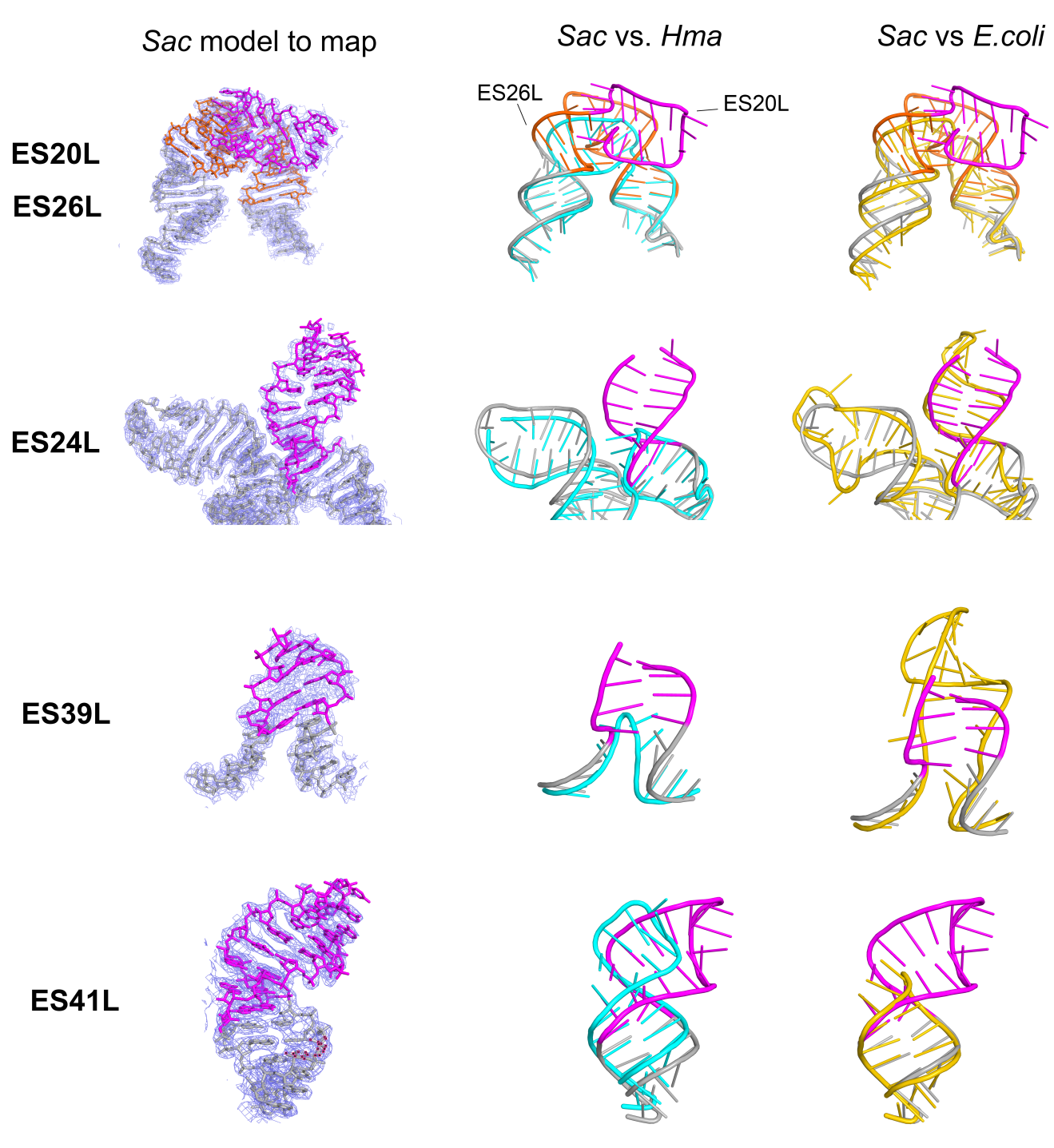
**

**Supplementary Figure 5. Cryo-EM density for ribosomal proteins and rRNA and secondary structures of rRNA**

(**A**) Cryo-EM map quality for ribosome subunits**.** Density map showed the fitting of ribosomal proteins, including eL30 (cyan), eL14 (pink or light blue), eL8 (blue), uS11 (orange), uS13 (yellow), and uS17 (purple).

(**B**) Distribution of density for rRNA expansion segments (red). Density is shown for ES9L.

(**C**) Density map showing the side chains of ribosomal proteins.

(**D**) Secondary structure diagram of *S. acidocaldarius* rRNA. The 5’ and 3’ region of 23S rRNA and 5S rRNA and 16S rRNA are shown.

(**E**) Representative ESs and VRs structure (yellow) compared to bacterial and eukaryotic homologous structures. ESs and VRs in the 3’ end and 5’ end of 23S rRNA are shown.

(**F**) Detailed EM map surrounding rRNA ES (magenta) and comparison with euryarchaeota *Haloarcula marismortui* (cyan) and bacteria *E.coli* (yellow)*.* ES4L, ES5L, ES9L, ES15L, ES20L, ES26L, ES24L, ES39L and ES41L are shown.


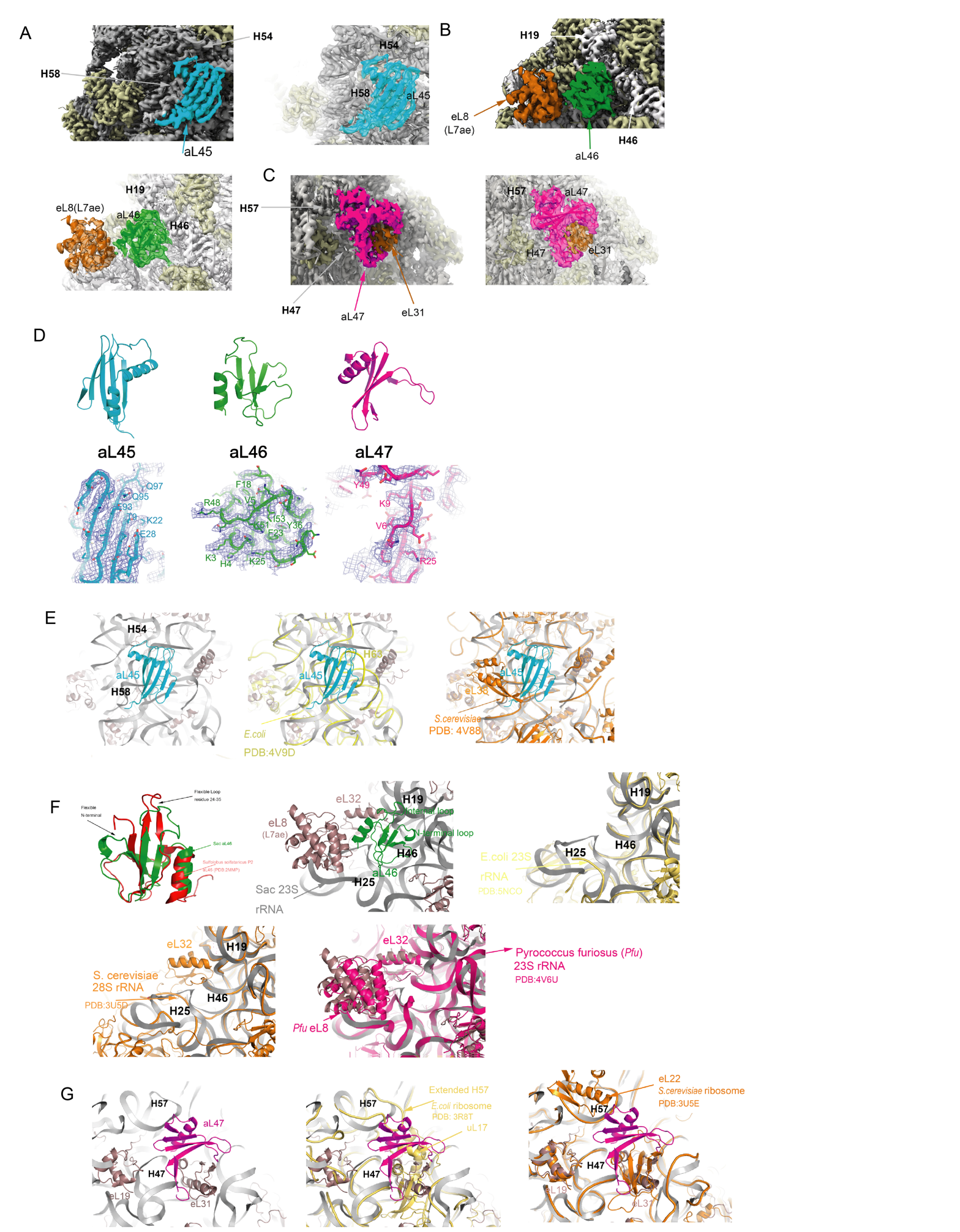


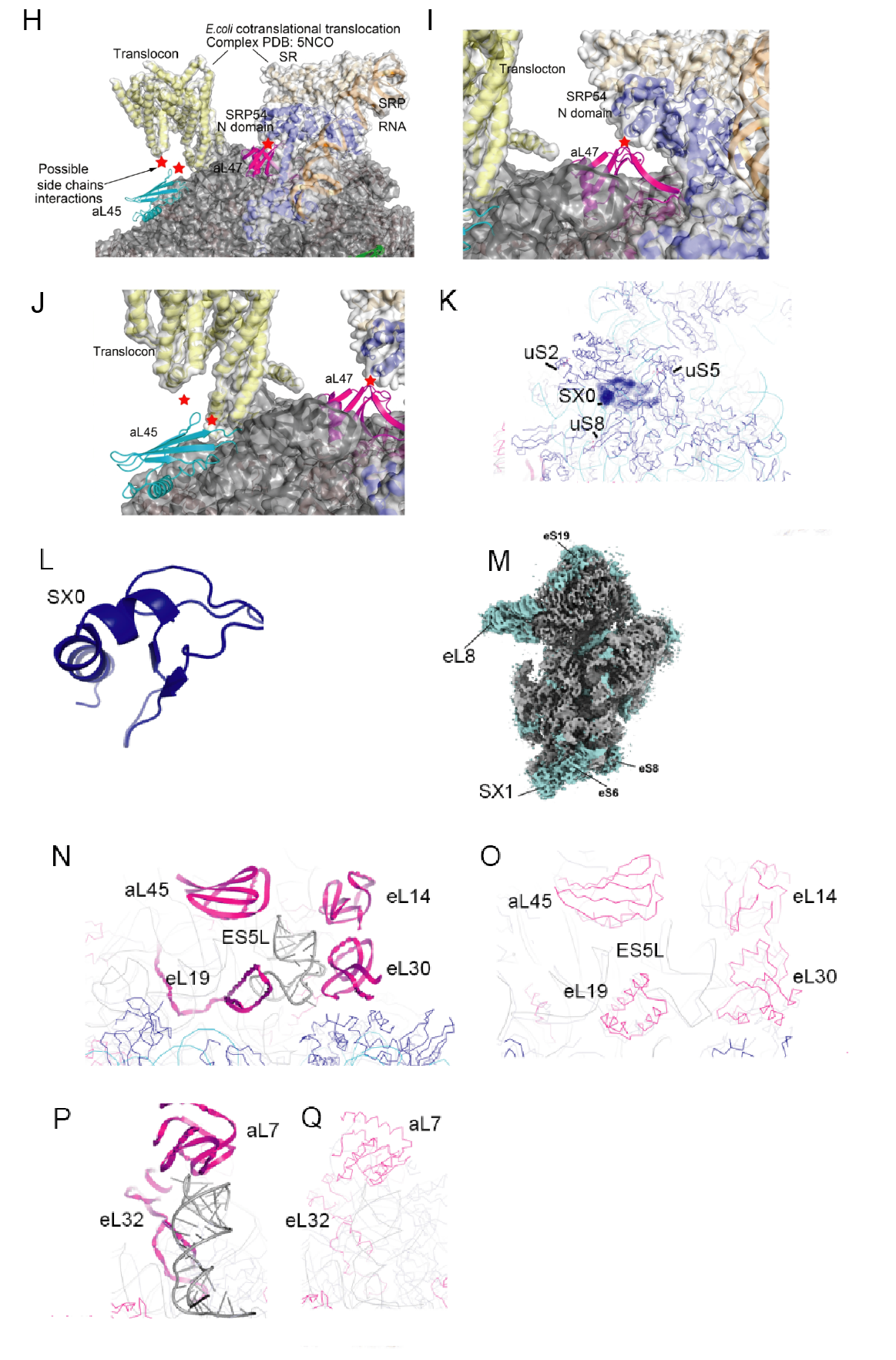


**
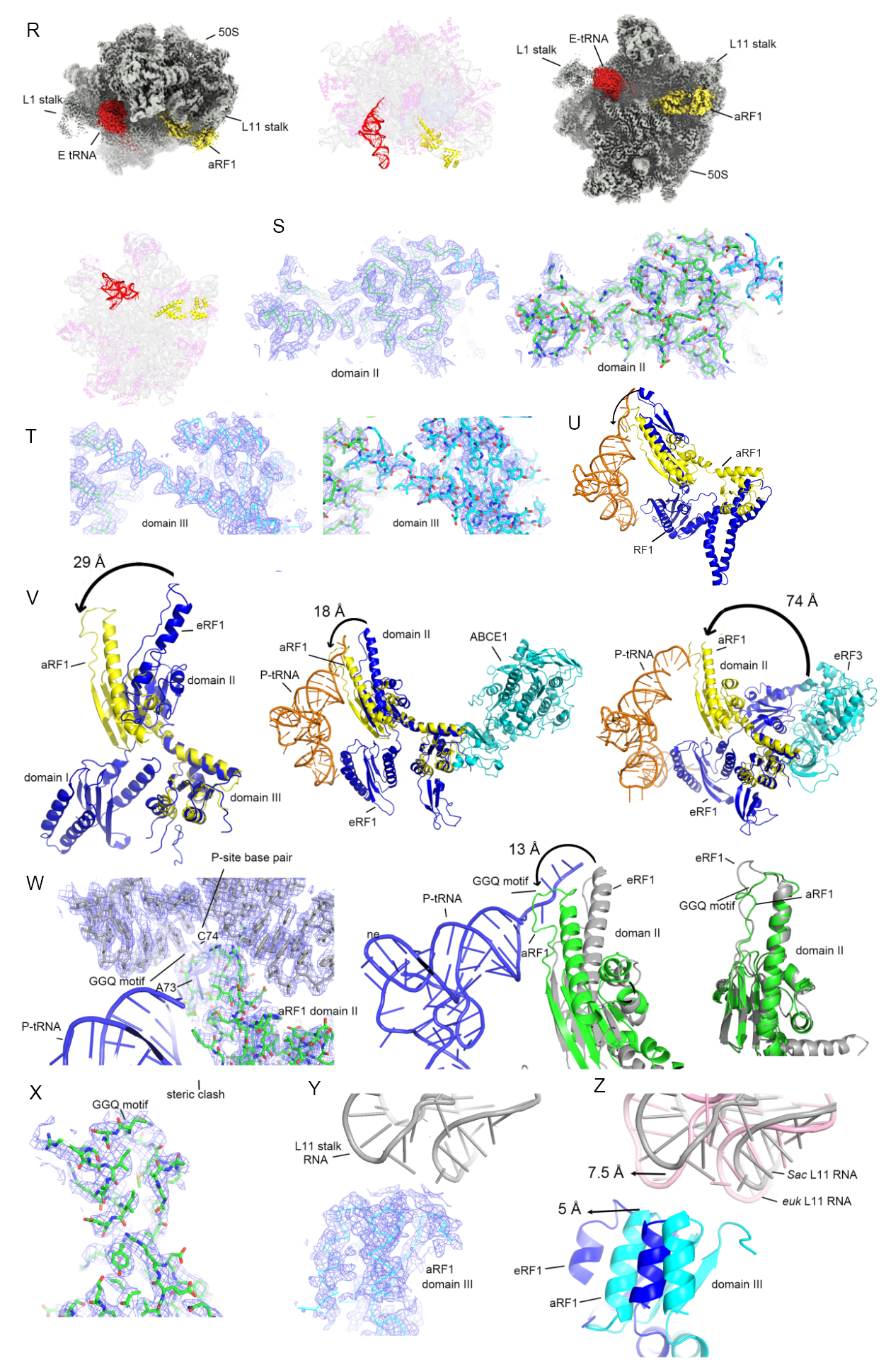
**

**
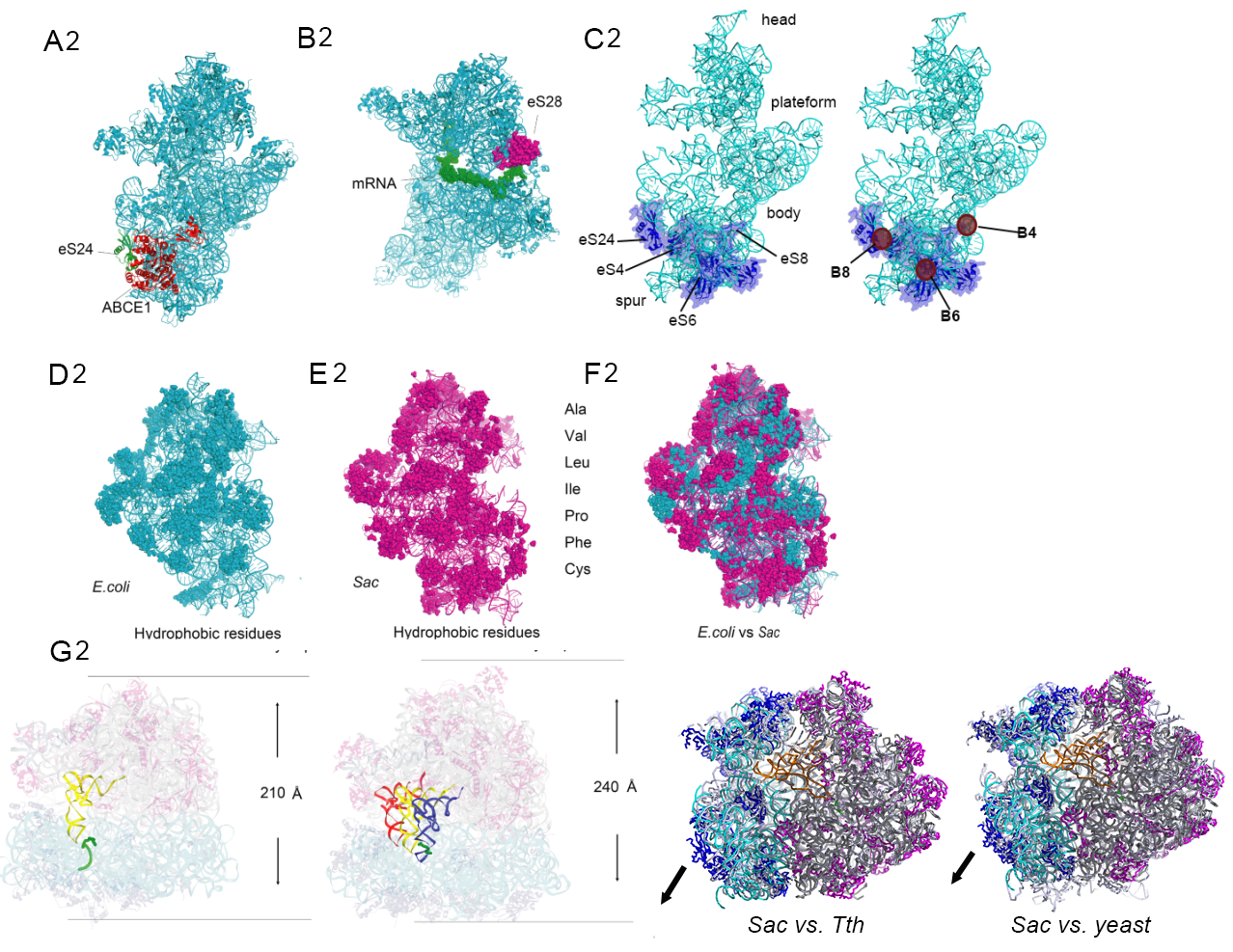
**

**Supplementary Figure 6. New ribosomal r-proteins and 50S-aRF1 structure**

(**A**). EM density indicates that aL45 (cyan) contacts H54 and H58 of 23S rRNA (gray).

(**B**). EM density indicates that aL46 (green) contacts eL8 (L7ae, orange) and H19 and H46 of 23S rRNA (gray).

(**C**). EM density indicates that aL47 (magenta) contacts eL31 (orange) and H47 and H57 of 23S rRNA (gray).

(**D**). The atomic models of aL45 (cyan), aL46 (green), and aL47 (purple). All of the side chains can be traced from the EM map for these three proteins.

(**E**). Binding of aL45 (cyan) on *Sac* LSU and comparison with *E. coli* (yellow) and *S. cerevisiae* (orange) ribosome. aL45 interacts with H54 and H58 of 23S rRNA (gray). The extended H63 in *E. coli* ribosome, which lacks aL45, partially overlaps with the position of aL45. The eL38 of *S. cerevisiae* ribosome is adjacent to and partially overlaps with aL45.

(**F**). Binding of aL46 (green) on *Sac* LSU and comparison with *E. coli* (yellow), *S. cerevisiae* (orange) and *Pfu* ribosomes (magenta). Superimposing structure of *Sac* aL46 (green, ribosome bound state) and the NMR spectroscopy solution structure of aL46 from *Sulfolobus solfataricus* P2 (red, ribosome-free state) reveals the flexibility of its N terminal residues and an internal loop (residues 24-35). The aL46 interacts with ribosomal protein eL32 and eL8 (L7ae) (light pink). It also contacts H19, H46, and H25 of 23S rRNA (gray). Interestingly, the flexible parts of this protein, the N terminal loop, and an internal loop undergo conformational changes compared to the ribosome-free state, when they form interaction with H46 and H19 of 23S rRNA, respectively. Structural comparison in the aL46 binding region of *E. coli, Sac, S. cerevisiae* and *Pfu* ribosomes suggests that H19, H46, and H25 are stabilized by eL8, aL46, and eL32 in *Sac* ribosome. In *E. coli* ribosome, these three proteins are absent. In *S. cerevisiae* ribosome, only eL32 is present. In *Pfu* ribosome, both eL32 and eL8 are present.

(**G**). Binding of aL47 (magenta) on *Sac* LSU and comparison with *E. coli* (yellow)and *S. cerevisiae* (orange) ribosomes. The aL47 contacts with H47 and H57 of 23S rRNA (gray). Also, it interacts with eL31 (light pink). Superimposing structure of *E. coli* ribosome and *Sac* ribosome indicates that the position of uL17 (yellow) and extended H57 in *E. coli* ribosome overlaps with the position of eL31 (light pink) and aL47. H57 is stabilized by aL47 in *Sac* ribosome whereas it is stabilized by eL22 (orange) in *S. cerevisiae* ribosome.

(**H-J**).The *Sac* aL47 (magenta) and aL45 (cyan) possibly form interactions with cotranslational translocation machinery (blue or orange or yellow). Docking bacterial SRP (PDB:5NCO) on *Sac* 50S ribosome based on 23S rRNA alignment. It indicates that aL47 can possibly interact with N-domain of SRP54 (blue) and aL45 can possibly interact with translocon (yellow).

(**K-L**) location and protein fold of protein SX0, which is also present in *Thermococcus kodakarensis* ribosome (*Tko*, PDB:6SKF) .

(**M**) location of new r-protein SX1.

(**N-Q**) Additional r-proteins around ESL5 versus *Tth* (N). Similar distribution around ESL5 versus yeast (O). Additional r-proteins around H25 versus *Tth* (P). aL7 overlap with r-RNA expansion segments versus yeast (Q).

(**R**) EM density and model showing binding of aRF1 (yellow) and E-site tRNA (red) to *Sac* 50S subunit (gray).

**(S)** EM density showing atomic model fitting of aRF1 domain II (green).

(**T**) EM density showing atomic model fitting of aRF1 domain III (cyan).

**(U)** Comparison of aRF1 (yellow) domain II’s orientation with that of bacterial RF1 suggests that domain II swings more and clash with P-site tRNA (PDB: 4V63, blue).

(**V**) Left: based on domain III docking, the long helix containing GGQ motif in domain II (yellow) swings 29 Å from ribosome free state (PDB:1DT9, blue).

Middle: based on domain III docking, aRF1 in Sac 50S (yellow) is compared with eRF1 in ribosome-eRF1-ABCE complex (PDB:3JAG; eRF1, blue; ABCE1, cyan) or in ribosome-eRF1-eRF3 complex (PDB:3J5Y; eRF1, blue; eRF3, cyan). Alignment on 23S rRNA shows that GGQ motif on aRF1 overlap with 3' end backbone (A73, C74) of P-site RNA in ribosome-eRF1-ABCE1 complex (PDB:3JAG).

Right: Structure comparison with termination complex (3JAG) shows that aRF1 domain II rotates 18 Å towards P-site tRNA. Compared with pre-termination complex (3J5Y), aRF1 domain II rotates as much as 74 Å.

(**W**) Cryo-EM density and model building suggests that the loop containing GGQ motif (green) in domain II of aRF1 undergo conformational changes compared to eRF1 (gray) in ribosome-eRF1-ABCE1 complex (PDB:3JAG, based on domain III alignment).

(**X-Z**) Cryo-EM density and structure alignment show that domain III's orientation (cyan) is coupled with movement of L11 stalk rRNA (gray). In the 80S-eRF1-ABCE1 complex (PDB:3JAG; eRF1, blue; L11 rRNA, light pink), L11 moves 7.5 Å towards P-site tRNA. This causes 5 Å movement of domain III of eRF1 compared with aRF1 domain III in *Sac* 50S-aRF1 complex. Such differences may be caused by termination factors's binding on 80S and 50S respectively.

**(A2)** eS24 (green) interacts with recycling factor ABCE1 (red, [Nürenberg-Goloub](https://www.embopress.org/action/doSearch?ContribAuthorRaw=N%C3%BCrenberg-Goloub%2C+Elina) et.al., *EMBO*, 2020. PDB: 6TMF)

**(B2)** eS28 (magenta sphere) interacts with mRNA (green sphere) in the E-site.

**(C2)** From subunit interface view, the location of additional r-protein eS24, eS8, eS6, and eS4 overlap with bacterial subunit bridges B4, B6 and B8

**(D2)** Distribution of hydrophobic residues (cyan, sphere) that buried inside *E.coli* 30S subunit.

**(E2)** Distribution of hydrophobic residues (magenta, sphere) that buried inside *Sac* 30S subunit. These residues include Ala, Val, Leu, Ile, Pro, Phe and Cys.

**(F2)** Overlapping the hydrophobic residues suggests that there are 31% more such residues inside *Sac* 30S compared to *E.coli*

**(G2)** Left: The diameter of *Sac* 70S ribosome (240 Å) is significantly longer than that of *E.coli* 70S ribosome (210 Å). Right:shift of *Sac* 30S towards solvent side versus *Tth* or yeast ribosome. Magenta, cyan, blue: Sac ribosome. Gray: *Tth* or yeast ribosome.

**
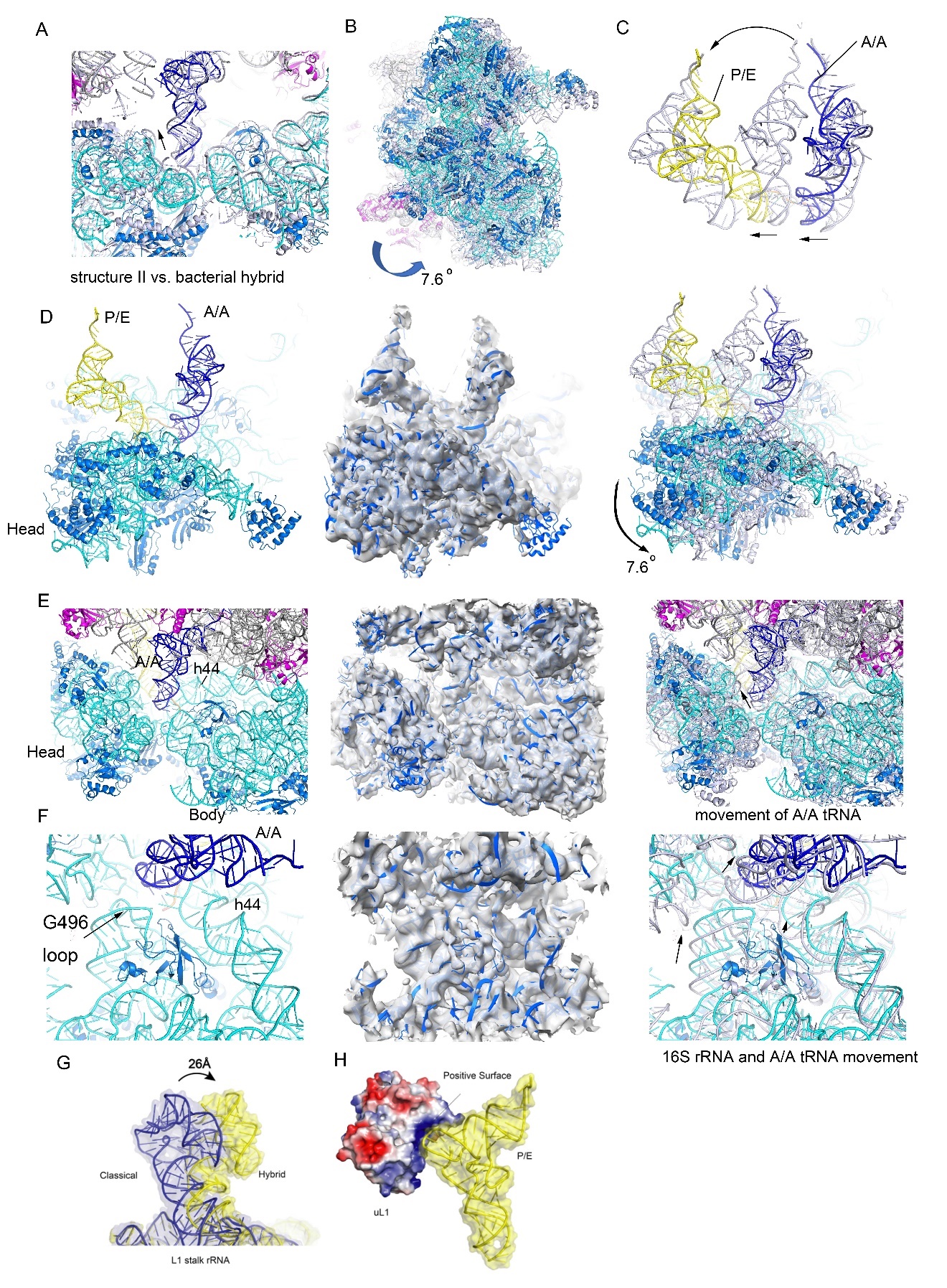
**

**Supplementary Figure 7. Overall structure of Sac ribosome in non-rotated state and A/A tRNA, hybrid P/E tRNA in rotated state ribosome.**

**(A)** Structural comparison of A/A tRNA and 30S subunit position in structural II versus bacterial ribosome with A/A and P/E tRNA (PDB:7SSN).

**(B)** 7.6º counterclockwise rotation of 30S subunit in structure II (hybrid) relative to 30S in structure I (classical).

**(C)** Positions and conformations of A/A and A/P tRNA in structure II versus A/A and P/P tRNA in structure I-A (based on 23 rRNA alignment).

**(D)** Comparing tRNA and 30S head domain in structure II with that in structure I-A.

**(E)** The view showing global movement of A/A tRNA and 30S subunit from structure I-A to structure II. Structure I with A/A tRNA is colored gray (based on 23 rRNA alignment)..

**(F)** A/A tRNA moving 12 Å along with G496 loop (G530 in bacterial ribosome) and h44 in the decoding center (based on 23 rRNA alignment)..

**(G)** In structure II, the distal part of L1 rRNA (blue) has moved inward by 20 Å to contact P/E tRNA (yellow).

**(H)** L1 interacts with P/E tRNA (yellow) elbow through a positive binding surface.

**
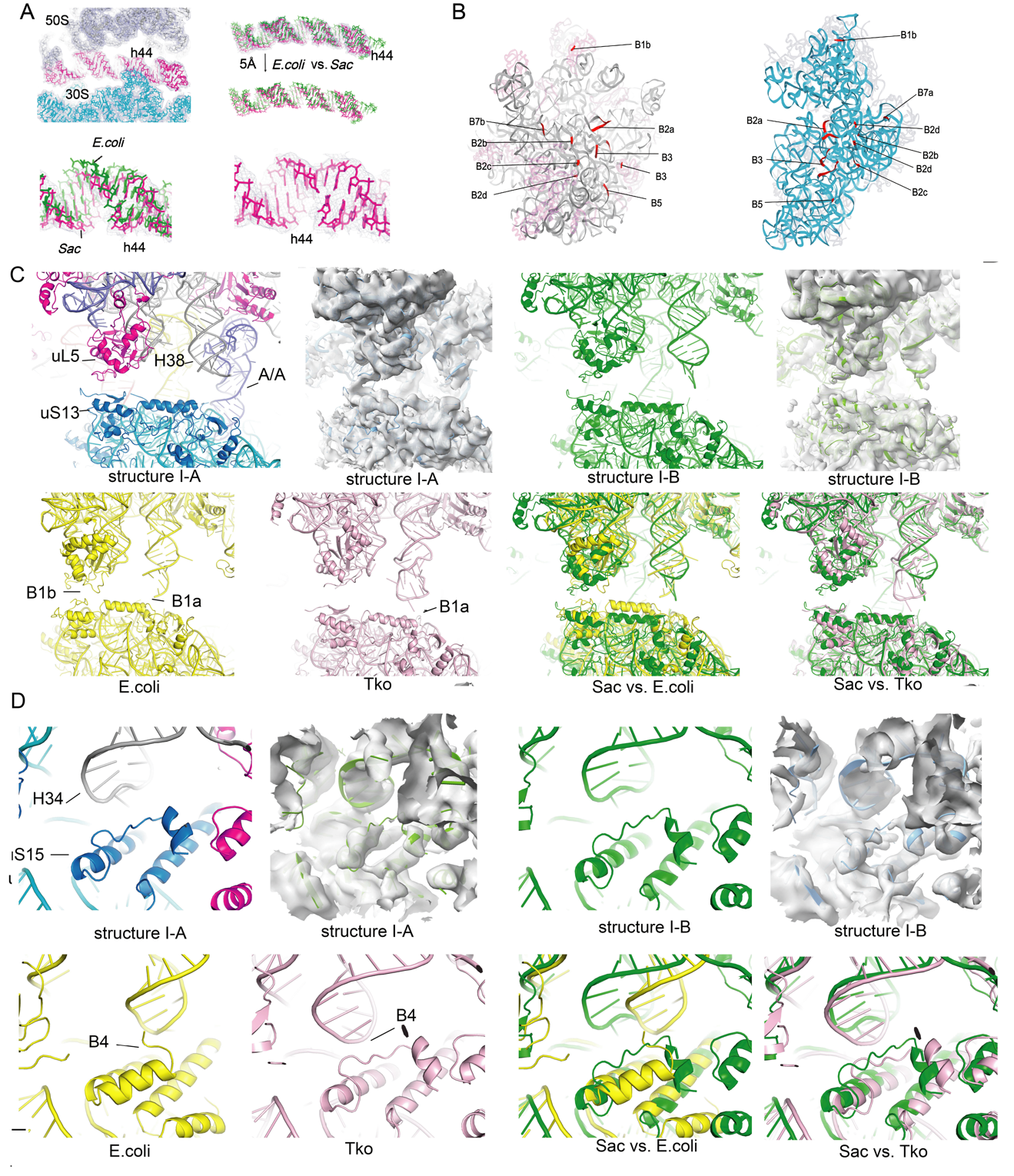
**

**
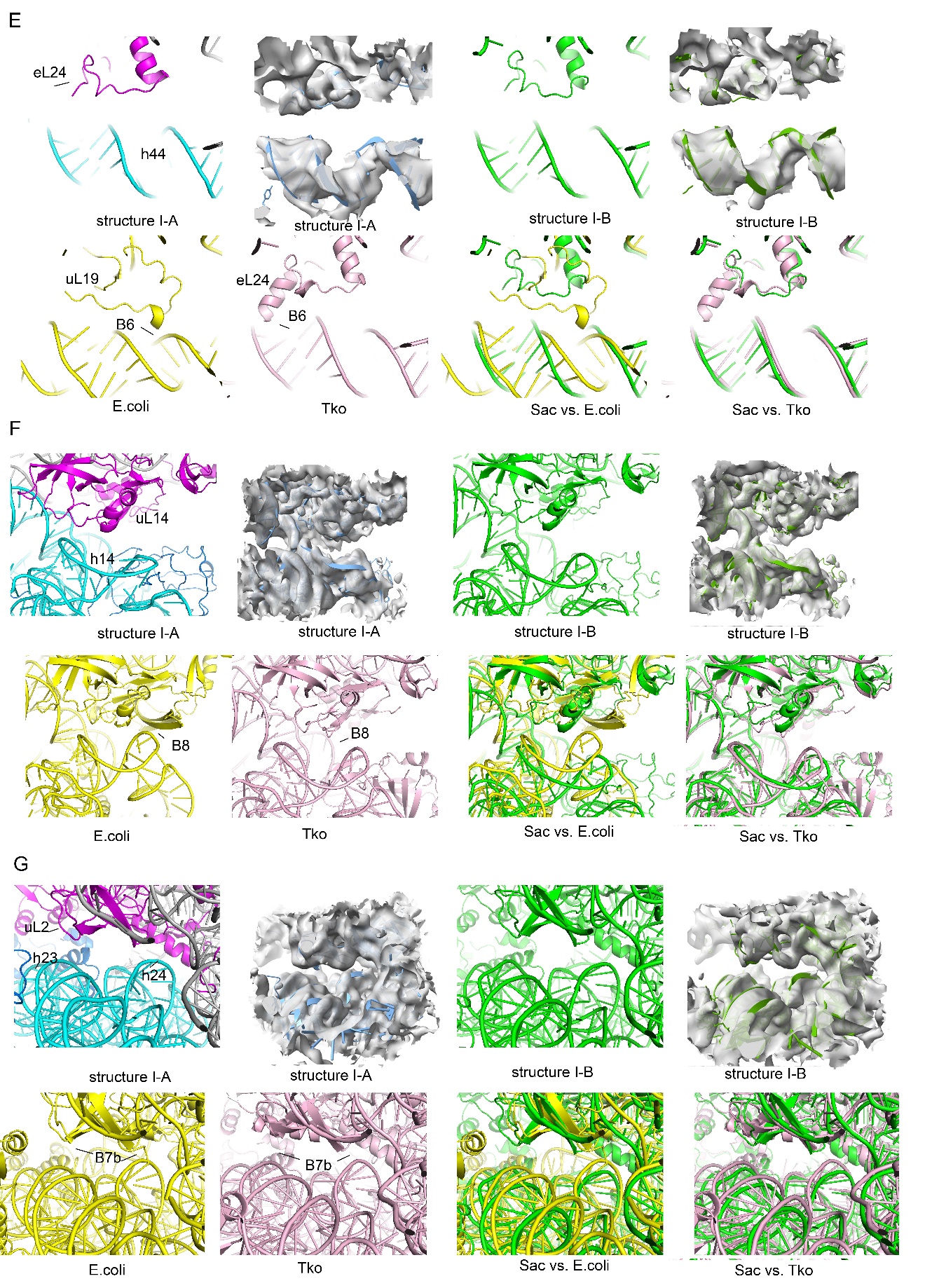
**

**Supplementary Figure 8. Conformational differences in h44 and bridge B1a, B4, B6, B8 and B7b between Sac and bacterial ribosomes (aligning on 23S rRNA).**

**(A)** Cryo-EM density of h44 of *Sac* ribosome (magenta) 16S rRNA and conformational differences compared with *E. coli* (green) and *Tth*. (yellow), ~ 5 Å shift of h44 from subunit interface in *Sac* compared with *E. coli* (PDB:4V9D) or *Tth* (PDB: 4V6F)

**(B)** Distribution of *Sac* 70S ribosomal inter-subunit bridges (structure I-A).

**(C)** Comparing subunit bridge B1a (bacterial, PDB:4V9D) components among *Sac*, *E.coli* and *Thermococcus kodakarensis* (*Tko*, PDB:6SKF). Structure I-A is shown in color (LSU r-proteins，magenta; SSU r-proteins, blue; 23S rRNA, black; 16S rRNA, cyan). Structure I-B is colored green. *E.coli* structure is colored yellow. *Tko* structure is colored light pink.

Overlap of bacterial B1a components on *Sac* cryo-EM map shows that the uS13 is 14 Å away from H38 (A-site finger). There are no bridge interactions in this region of *Sac*.

**(D)*.*** The *Sac* uS15 backbone is 6 Å from H34 of 23S rRNA. Thus, there is no bridge B4 in *Sac*.

**(E)** Movement of *Sac* h44. and the structural differences between eL24 and uL19 lead to the elimination of bridge B6 in *Sac*.

**(F)** The uL14 is 7 Å from h14 of 16S rRNA. Thus, there is no bridge B8 in *Sac*.

**(G)** The 3-4 Å movements of h23 and h24 lead to the elimination of bacterial bridge B7b in *Sac*. The uL2 backbone is 9 Å away from h23 and 5 Å away from the h24 of 16S rRNA. Thus, there is no bridge B7b in *Sac*.


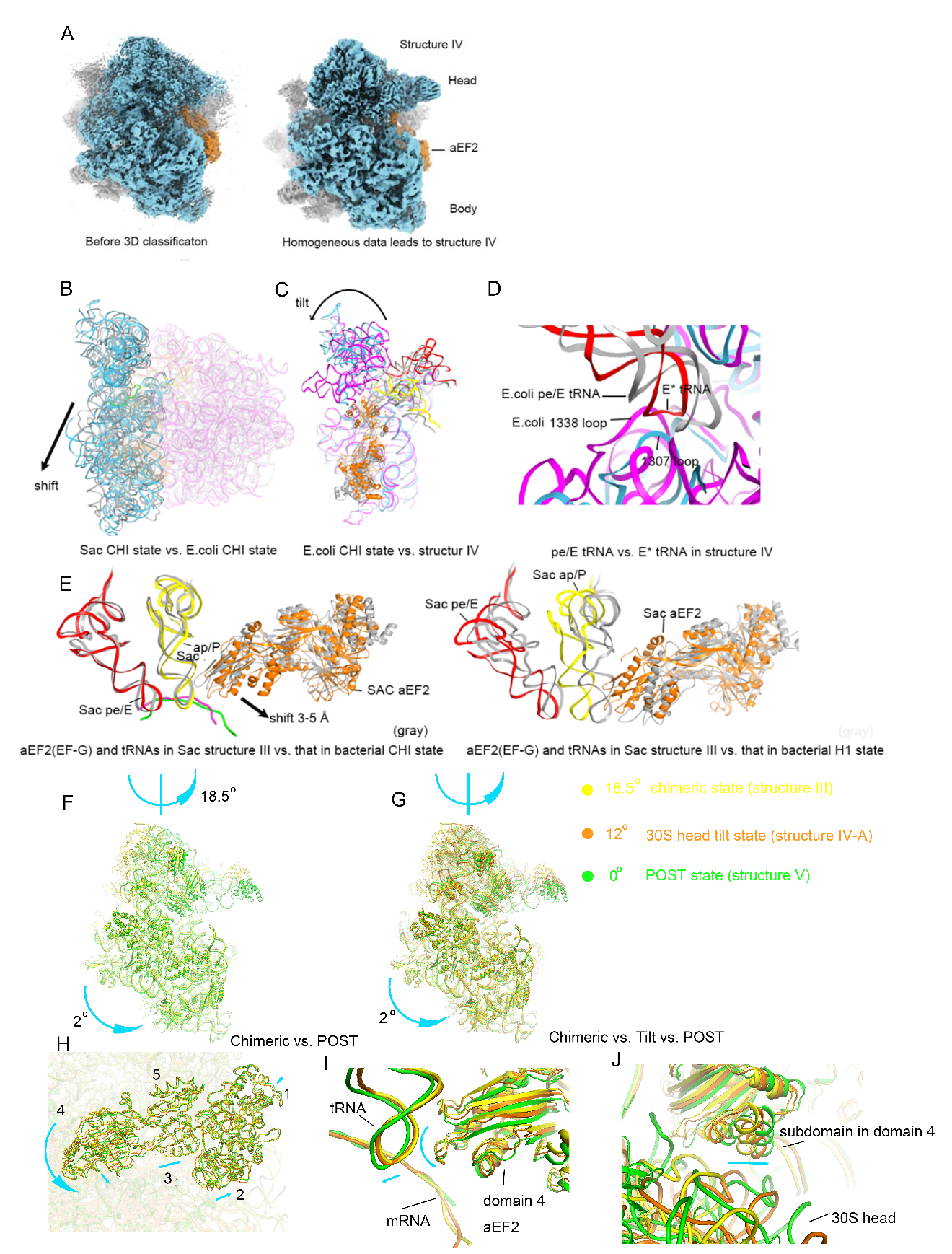


**Supplementary Figure 9. aEF2-ribosome complexes comparison (structure III, IV-A, IV-B, and V)**

**(A)** Cryo-EM maps showing the data heterogeneity for sample 3 focused on the 30S head domain. Left: The 3D classification based on entire 30S subunit resulted in fuzzy density on 30S head. Right: The 3D classification based on 30S head lead to well-resolved density on 30S head (structure IV is shown; 50S, gray; 30S, cyan; aEF2, orange).

(**B-E**) Structure comparison of *Sac* translocation complexes with bacterial translocation complex solved by time-resolved cryo-EM:

(**B**) Alignment of 23S rRNA shows that 30S in *Sac* structure III (CHI state, cyan) shifts towards solvent side compared with 30S in bacterial CHI state (PDB:7PJY, gray).

(**C-D**) Comparison between *Sac* structure IV-A and bacterial CHI state ribosome (PDB:7PJY, magenta) indicates *Sac* 30S (cyan) tilt to release E* tRNA (red).

(**E**) Left: Inspecting tRNAs (yellow or red) and aEF2 (orange) position in *Sac* structure III versus that in bacterial CHI state (gray) shows general shifts of tRNAs, aEF2 and mRNA which are caused by 30S shift. This is consistent with the finding in classical *Sac* 70S complex, in which tRNAs shift along with 30S position.

Right: comparison of ligands positions in *Sac* structure III (CHI state, colored) with that in H1 state (gray) in bacterial ribosome. We did not capture H1 state with aEF2 binding. It is likely that H1 state (EF-G engaged) is too transient, and only can by captured by time-resolved cryo-EM.

**(F)** 30S subunit in structure III is compared with that in structure V.

**(G)** Structural comparison of 30S subunit in structure III, IV-A and V. The 30S in structure III undergo 2º counterclockwise rotation relative to 50S. 30S head swiveled 18º, 12º and 0º respectively in structure III, IV and V.

**(H)** Conformational changes in domain 1,2,3,4,5 of aEF2 among structure III, IV-A, and V. The trend is that aEF2 gradually move apart from translocated tRNA.

**(I)** Domain 4 of aEF2 gradually moves downwards as tRNA proceeds to P-site.

**(J)** The subdomain in domain 4 of aEF2 gradually moves as 30S head domain swives to different state.

**Supplementary Table 1. Statistics of Cryo-EM structural determination and model refinement.**
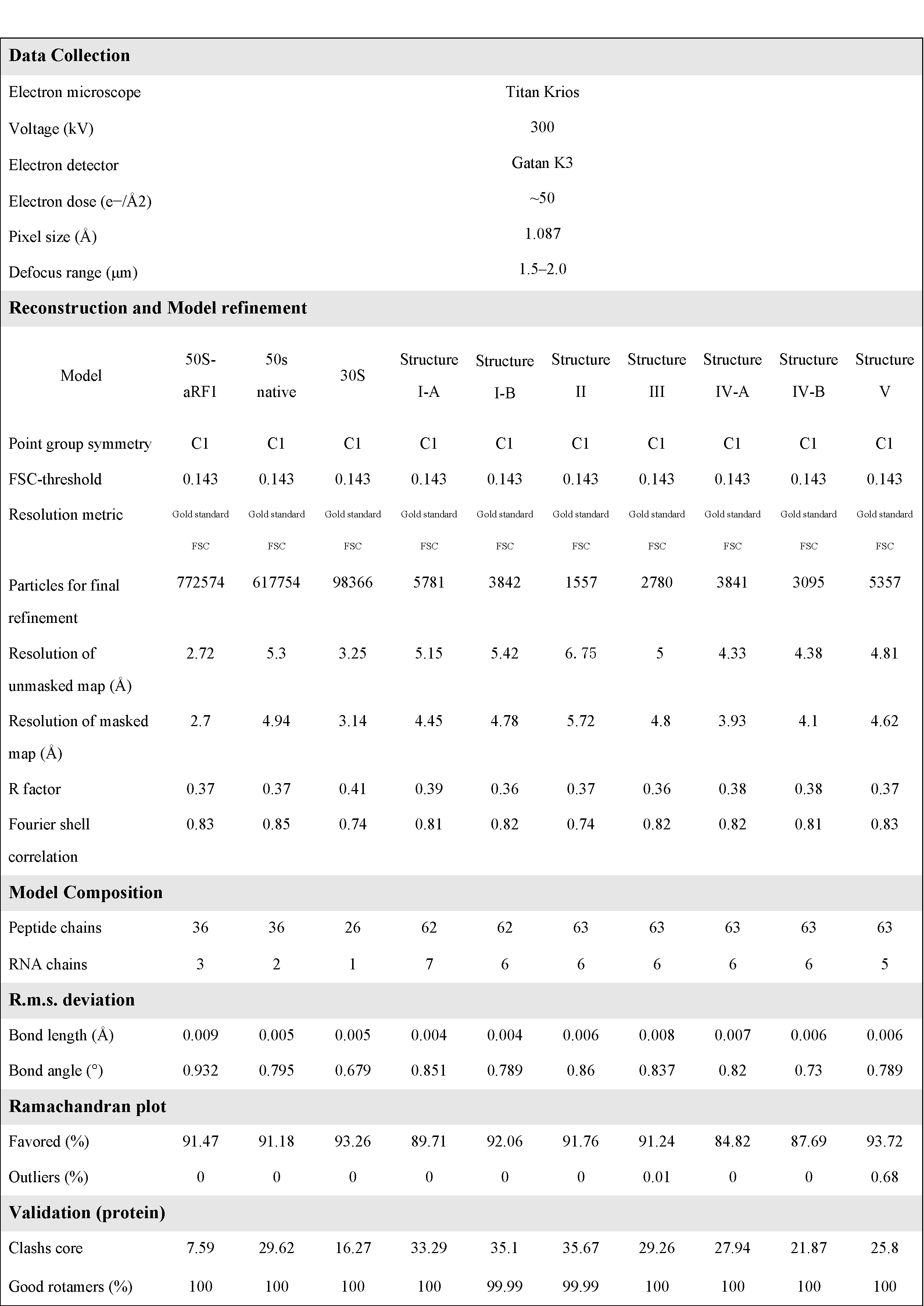


1. Cukras, A.R., Southworth, D.R., Brunelle, J.L., Culver, G.M. and Green, R. (2003) Ribosomal proteins S12 and S13 function as control elements for translocation of the mRNA:tRNA complex. *Mol Cell*, **12**, 321-328.

2. Schmeing, T.M., Voorhees, R.M., Kelley, A.C., Gao, Y.G., Murphy, F.V.t., Weir, J.R. and Ramakrishnan, V. (2009) The crystal structure of the ribosome bound to EF-Tu and aminoacyl-tRNA. *Science*, **326**, 688-694.

3. Selmer, M., Dunham, C.M., Murphy, F.V.t., Weixlbaumer, A., Petry, S., Kelley, A.C., Weir, J.R. and Ramakrishnan, V. (2006) Structure of the 70S ribosome complexed with mRNA and tRNA. *Science*, **313**, 1935-1942.

4. Wimberly, B.T., Brodersen, D.E., Clemons, W.M., Jr., Morgan-Warren, R.J., Carter, A.P., Vonrhein, C., Hartsch, T. and Ramakrishnan, V. (2000) Structure of the 30S ribosomal subunit. *Nature*, **407**, 327-339.

5. Yusupov, M.M., Yusupova, G.Z., Baucom, A., Lieberman, K., Earnest, T.N., Cate, J.H. and Noller, H.F. (2001) Crystal structure of the ribosome at 5.5 A resolution. *Science*, **292**, 883-896.

6. Liu, Q. and Fredrick, K. (2016) Intersubunit Bridges of the Bacterial Ribosome. *J Mol Biol*, **428**, 2146-2164.

7. Reblova, K., Razga, F., Li, W., Gao, H., Frank, J. and Sponer, J. (2010) Dynamics of the base of ribosomal A-site finger revealed by molecular dynamics simulations and Cryo-EM. *Nucleic Acids Res*, **38**, 1325-1340.

8. Liu, Q. and Fredrick, K. (2013) Contribution of intersubunit bridges to the energy barrier of ribosomal translocation. *Nucleic Acids Res*, **41**, 565-574.

9. Goddard, T.D., Huang, C.C., Meng, E.C., Pettersen, E.F., Couch, G.S., Morris, J.H. and Ferrin, T.E. (2018) UCSF ChimeraX: Meeting modern challenges in visualization and analysis. *Protein Sci*, **27**, 14-25.

10. Emsley, P. and Cowtan, K. (2004) Coot: model-building tools for molecular graphics. *Acta Crystallogr D*, **60**, 2126-2132.

11. Drozdetskiy, A., Cole, C., Procter, J. and Barton, G.J. (2015) JPred4: a protein secondary structure prediction server. *Nucleic Acids Research*, **43**, W389-W394.

12. Kelley, L.A., Mezulis, S., Yates, C.M., Wass, M.N. and Sternberg, M.J.E. (2015) The Phyre2 web portal for protein modeling, prediction and analysis. *Nat Protoc*, **10**, 845-858.

13. Stein, N. (2008) CHAINSAW: a program for mutating pdb files used as templates in molecular replacement. *J Appl Crystallogr*, **41**, 641-643.

14. Waterhouse, A., Bertoni, M., Bienert, S., Studer, G., Tauriello, G., Gumienny, R., Heer, F.T., de Beer, T.A.P., Rempfer, C., Bordoli, L. *et al.* (2018) SWISS-MODEL: homology modelling of protein structures and complexes. *Nucleic Acids Research*, **46**, W296-W303.
